# Inhibition of the transcriptional kinase CDK7 overcomes therapeutic resistance in HER2-positive breast cancers

**DOI:** 10.1038/s41388-019-0953-9

**Published:** 2019-08-28

**Authors:** Bowen Sun, Seth Mason, Robert C. Wilson, Starr E. Hazard, Yubao Wang, Rong Fang, Qiwei Wang, Elizabeth S. Yeh, Meixiang Yang, Thomas M. Roberts, Jean J. Zhao, Qi Wang

**Affiliations:** 1 The First Affiliated Hospital, Biomedical Translational Research Institute and School of Pharmacy, Jinan University, Guangzhou, 510632 China; 2 Department of Pathology and Laboratory Medicine, Medical University of South Carolina, Charleston, SC 29425 USA; 3 Computational Biology Resource Center, Medical University of South Carolina, Charleston, SC 29425 USA; 4 Department of Cancer Biology, Dana-Farber Cancer Institute, Boston, MA 02115 USA; 5 Department of Biological Chemistry and Molecular Pharmacology, Harvard Medical School, Boston, MA 02115 USA; 6 Department of Pathology, Zhejiang Provincial Key Laboratory of Pathophysiology, Ningbo University School of Medicine, Ningbo, 315211 China; 7 Department of Cell and Molecular Pharmacology and Experimental Therapeutics, Medical University of South Carolina, Charleston, SC 29425 USA

**Keywords:** Diagnostic markers, Breast cancer

## Abstract

Resistance of breast cancer to human epidermal growth factor receptor 2 (HER2) inhibitors involves reprogramming of the kinome through HER2/HER3 signaling via the activation of multiple tyrosine kinases and transcriptional upregulation. The heterogeneity of induced kinases prevents kinase targeting by a single kinase inhibitor and presents a major challenge to the treatment of therapeutically recalcitrant HER2-positive breast cancers (HER2+ BCs). As a result, there is a critical need for effective treatment that attacks the aberrant kinome activation associated with resistance to HER2-targeted therapy. Here, we describe a novel treatment strategy that targets cyclin-dependent kinase 7 (CDK7) in HER2 inhibitor-resistant (HER2iR) breast cancer. We show that both HER2 inhibitor-sensitive (HER2iS) and HER2iR breast cancer cell lines exhibit high sensitivity to THZ1, a newly identified covalent inhibitor of the transcription regulatory kinase CDK7. CDK7 promotes cell cycle progression through inhibition of transcription, rather than via direct phosphorylation of classical CDK targets. The transcriptional kinase activity of CDK7 is regulated by HER2, and by the receptor tyrosine kinases activated in response to HER2 inhibition, as well as by the downstream SHP2 and PI3K/AKT pathways. A low dose of THZ1 displayed potent synergy with the HER2 inhibitor lapatinib in HER2iR BC cells in vitro. Dual HER2 and CDK7 inhibition induced tumor regression in two HER2iR BC xenograft models in vivo. Our data support the utilization of CDK7 inhibition as an additional therapeutic avenue that blocks the activation of genes engaged by multiple HER2iR kinases.

## Introduction

The human epidermal growth factor receptor 2 (HER2, also known as ERBB2) [1, 2] is constitutively activated by overexpression or gene amplification in ~15–20% of human breast cancers [3]. HER2 serves as a bona fide oncogene that confers a more aggressive tumor phenotype and is associated with an increased rate of recurrence and mortality [1]. Trastuzumab (Herceptin) and lapatinib, two approved therapies for HER2-positive breast cancers (HER2+ BCs), have significantly improved the outcomes for HER2+ BC patients and have opened up an era of cancer treatment in this disease via targeted therapy [2, 3]. Unfortunately, nearly all patients either fail to respond to targeted therapies or have an initial response followed by the ultimate development of resistance. Diverse intrinsic or acquired mechanisms of resistance to HER2-targeted therapy have been described, such as the activation of parallel signaling pathways, hyperactivation of the downstream phosphoinositide 3-kinase-protein kinase B (PI3K-AKT) pathway, and adaptive kinome reprogramming [4–14]. As such, the use of targeted therapeutic agents that inhibit any single pathway would be undermined by activation of compensatory pathways [15]. Thus, a strategy that targets the transcription of key resistance elements in a more general manner might be more effective in HER2 inhibitor-resistant (HER2iR) breast cancers.

The cyclin-dependent kinase 7 (CDK7) is the catalytic subunit of the well-known CDK-activating kinase (CAK), a kinase complex that catalyzes phosphorylation of the T-loops and thereby activating multiple cyclin-associated kinases including CDK1, CDK2, CDK4, and CDK6. CAK also complexes with the core human transcription factor II (TFIIH) basal transcription complex and activates RNA polymerase II (RNA Pol II) by serine phosphorylation of the repetitive C-terminal domain (CTD) of its largest subunit. This phosphorylation permits RNA Pol II escape from promoters, thus facilitating the subsequent elongation of the resulting transcripts [5]. In addition, CDK7 has been shown to specifically modulate estrogen receptor (ER) activity through serine 118 (Ser118) phosphorylation [16]. THZ1, a relatively selective covalent inhibitor of CDK7, effectively inhibits the growth of several cancer types such as T-cell acute lymphoblastic leukemia, MYCN-amplified neuroblastoma, and small cell lung cancer [17–19]. More recently, THZ1 was shown to inhibit the growth of triple-negative breast cancer (TNBC) and ER+ BC) cells by inhibiting phosphorylation of the RNA Pol II CTD [20] and Ser118 of the ER [21, 22], respectively. The function of this kinase in other subtypes of breast cancer has not yet been elucidated. We previously reported the overexpression of genes regulating the cell division cycle in HER2+ mammary tumors results in resistance to HER2-targeted therapy. Blockage of cell cycle progression by CDK4/6 inhibitors resensitizes these cells to HER2 inhibitors [9], raising the question of whether inhibition of CDK7, a CDK4 activator, might overcome resistance to HER2 inhibitors. Here, we show that dual treatment combining HER2-targeted therapy with the CDK7 inhibitor THZ1 strongly inhibits HER2+ BC cell growth and increases apoptosis in cancer cells that exhibit resistance to HER2-targeted therapies. Unexpectedly, CDK7 inhibition does not appear to promote cell cycle progression through phosphorylation of CDKs in these cells. Rather, our data indicates that CDK7 primarily functions as a TFIIH-associated kinase in HER2+ BC. We also show that SHP2, and PI3K/AKT, key downstream signaling components of multiple receptor tyrosine kinases (RTKs), regulate the phosphorylation of RNA pol II CTD via activation of CDK7, suggesting that targeting CDK7 may provide an elegant mechanism by which to block reactivation of multiple kinase pathways in breast cancer resistant to HER2-targeted therapies.

## Results

### HER2+ breast cancer cells are susceptible to CDK7 inhibition independent of hormone receptor status

**Fig. 1:**
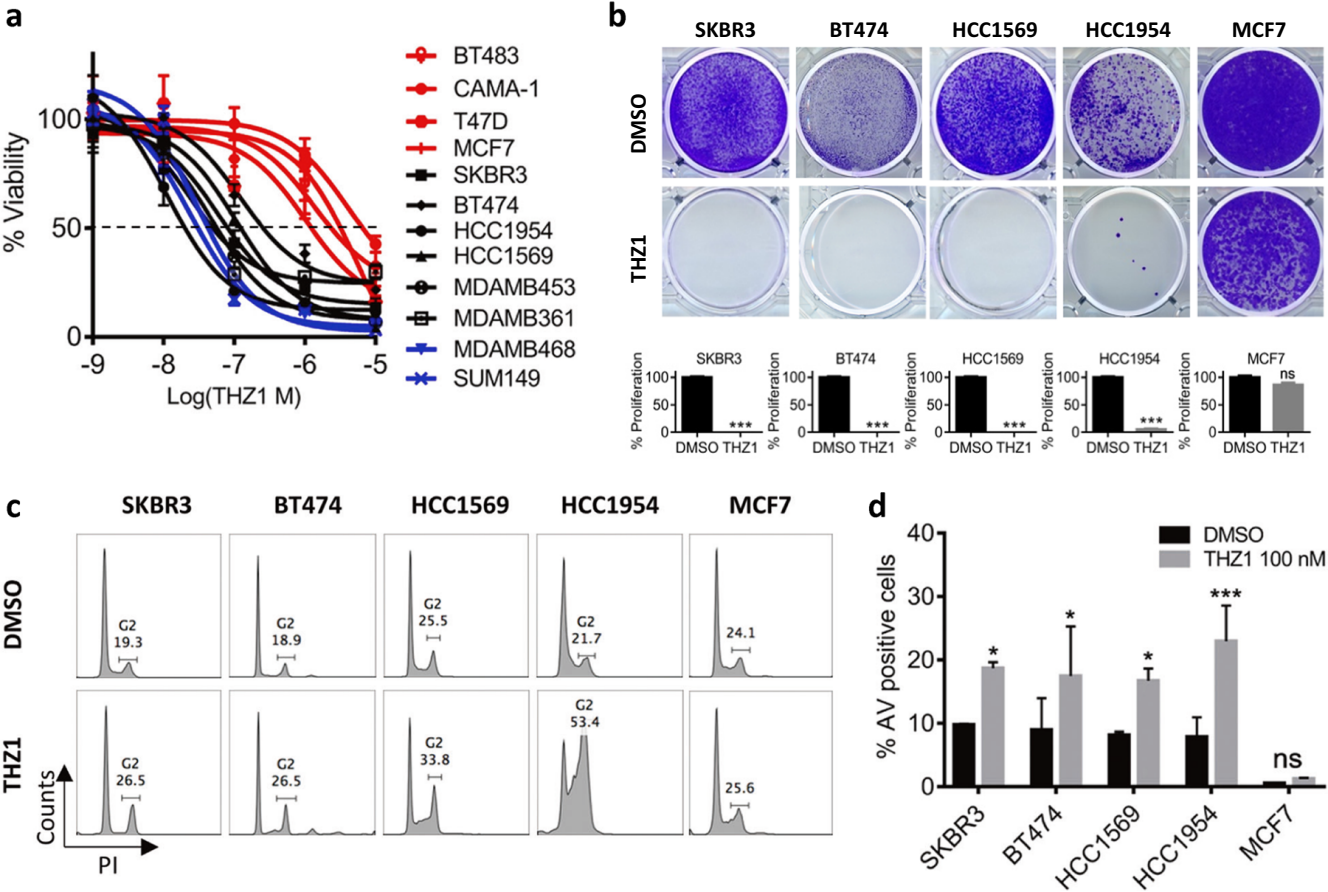
HER2+ breast cancer cells are susceptible to CDK7 inhibition independent of hormone receptor status. **a** Dose-response curve of breast cancer cell viability after treatment with increasing concentrations of THZ1 for 72 h. Percent viability relative to that of DMSO-treated cells is shown. Data represent mean ± SD of replicates from two or three independent experiments. Red, ER/PR+; black, HER2+ and ER/PR+; blue, TNBC. **b** Crystal violet staining of cells and quantification of cell growth. Data represent mean ± SEM of three replicates. ***p < 0.001; ns, not significant (one-way ANOVA). **c** Cell cycle analysis of cells treated with vehicle control (DMSO) or THZ1 (100 nM) for 24 h. **d** Flow cytometric analysis of annexin V-positive cells. Each cell line was treated with THZ1 (100 nM) for 24 h and stained with FITC-conjugated annexin V. Data represent mean ± SEM from three independent experiments, *p < 0.05; ns, not significant (one-way ANOVA)

We first investigated whether CDK7 inhibition affected the viability of HER2+ tumors cells with differing ER and progesterone receptor (PR) status. Cell lines tested included two HER2 inhibitor-sensitive (HER2iS) cell lines (SKBR3 and BT474) and four HER2iR cell lines (MDAMB453, HCC1954, HCC1569, and MDAMB361). BT474 and MDAMB453 cells were positive for both HER2 and either ER or PR [7, 9]. We also included two TNBC cell lines (MDAMB468, SUM149) and four ER/PR+ breast cancer cell lines (BT483, CAMA-1, MCF7, and T47D) as controls for the response to THZ1 [19, 20, 22]. TNBC cells were previously reported to be more sensitive to THZ1 treatment than ER/PR cells [20]. All HER2+ BC cell lines tested demonstrated high susceptibility to THZ1, with cell viability effectively suppressed at low nanomolar concentrations (average IC50 = 60 nM), similar to the effects seen in TNBC (Fig. 1a). ER/PR+ cells were less sensitive to THZ1, (average IC50 > 1 μM), consistent with a previous report [20]. We further evaluated the effect of THZ1 on long-term colony formation in HER2+ BC cells chronically exposed to a low dose (40 nM) of THZ1 (Fig. 1b). THZ1 efficiently inhibited colony formation in all HER2+ cells, but not in ER/PR+ MCF7 cells. Flow cytometric analyses of the cell cycle showed that, in contrast to TNBC and ER/PR cells [20], HER2+ cells treated with THZ1 arrested the G2/M phase of the cell cycle at 24 h of treatment (Fig. 1c). Moreover, THZ1 treatment led to profound induction of apoptosis in HER2+ cells, but not in ER/PR cells (MCF7) (Fig. 1d and Supplementary Fig. S1). Together, these data indicate that THZ1 is highly cytotoxic not only in TNBC cells, but also in HER2+ BC cells, regardless of their sensitivity to HER2 inhibitors and ER/PR status.

### Inhibition of CDK7 impacts transcription and cell cycle progression in HER2+ breast cancer cells

**Fig. 2:**
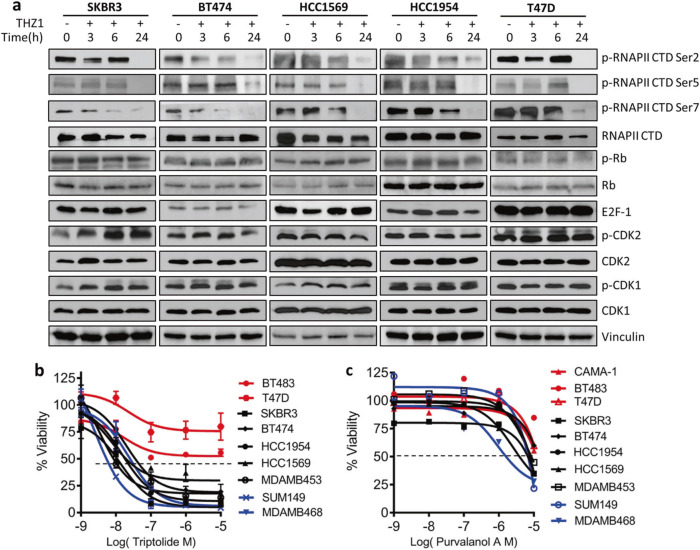
Inhibition of CDK7 impacts transcription and cell cycle progression in HER2+ breast cancer cells. **a** Cells were treated with vehicle control (DMSO) or THZ1 (100 nM) at 0, 3, 6, or 24 h before immunoblotting using the indicated antibodies. **b**, **c** Dose-response curves of viability of breast cancer cells after treatment with increasing concentrations of triptolide or purvalnolol for 72 h. Percent viability relative to that of DMSO-treated cells is shown. Data represent mean ± SD of replicates from two or three independent experiments

CDK7 regulates RNA Pol II-mediated transcriptional initiation and pausing, in addition to its effects on transcript elongation through its CAK activity working via other transcriptional CDKs [23–25]. We observed a decrease in the initiation-associated phosphorylation of serine 5/7 (S5, S7) and in the elongation-associated phosphorylation of S2 of Pol II in both HER2+ and ER/PR+ cells treated with THZ1. The decrease in Pol II phosphorylation following THZ1 treatment in HER2+ cells was associated with increased levels of cleaved poly ADP ribose polymerase (PARP), a marker of apoptotic cell death (Fig. 2a, Supplementary Fig. S2a, b). CDK7 also stimulates cell cycle progression by activating CDK1 and CDK2 through its T-loop phosphorylation function [26]. However, we did not observe a time-dependent decrease in T-loop phosphorylation of CDK1, CDK2, or other proteins involved in cell cycle regulation (pRB and E2F) in HER2+ cells (Fig. 2a and Supplementary Fig. S2c). Consistent with these results, the Pol II inhibitor triptolide appeared to show selectivity for HER2+ cells compared with purvalanol, which primarily targets cell cycle CDKs (Fig. 2b, c). Changes in phosphorylation of ER (Ser118) previously reported upon treatment with THZ1 were not observed in two HER2+/ER+ cell lines (BT474 and MDAMB453, Supplementary Fig. S2d). Thus, THZ1 appears to exert its cytotoxic effects through inhibition of transcription rather than via phosphorylation of classic CDKs or ER, suggesting that CDK7 plays a primary role in transcription in HER2+ BC cells.

### Modulation of CDK7/RNA pol II activity by HER2 signaling pathways

**Fig. 3:**
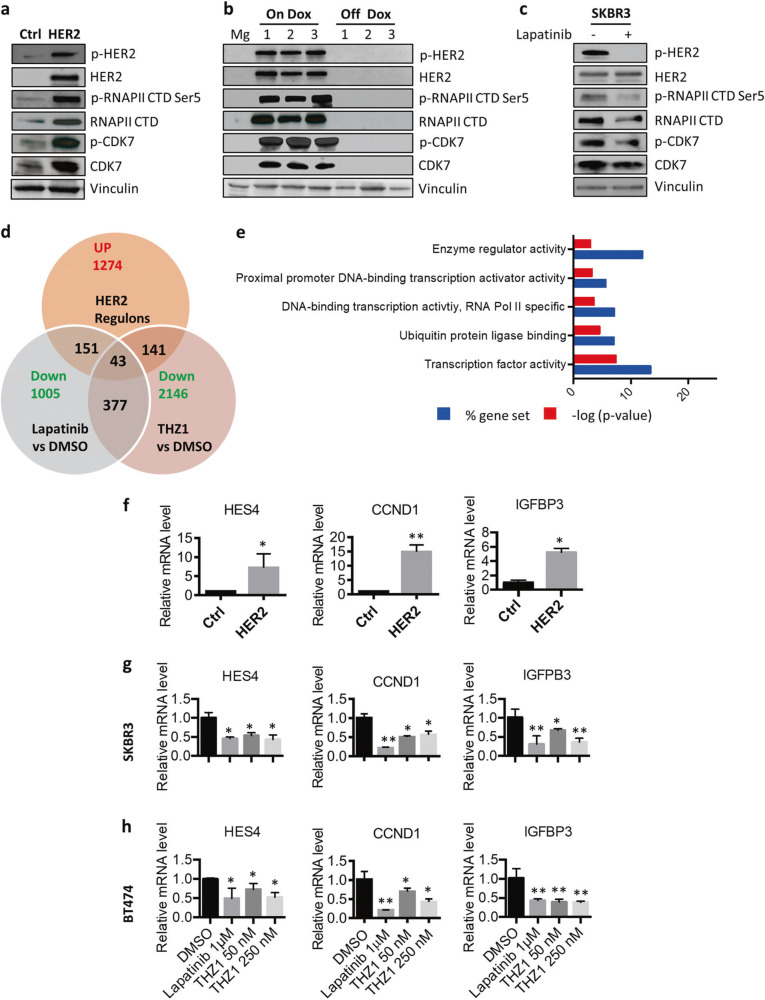
HER2 modulates CDK7 activity and CDK7-dependent gene transcription. **a** Effect of ectopic expression of human wild-type HER2 on protein expression in immortalized human mammary epithelial (HMEC) cells. **b** Protein expression after de-induction of HER2 expression in HER2+ mouse mammary tumors (Dox off). **c** SKBR3 cells were treated with vehicle control (DMSO) or lapatinib (1 μM) for 24 h before immunoblotting using the indicated antibodies. **d** Overlap of genes that were upregulated by HER2 in HMECs and inhibited by lapatinib and/or THZ1 in HER2+ BCs. **e** Gene oncology analyses of HER2 up-regulons inhibited by THZ1 expression in HMEC cells. **f** Q_RT-PCR analysis mRNA expression for HMEC-HER2 cell, compared the vector control cell HMEC-pBABE. Data represent mean ± SD (*n* = 3). *p *<* 0.05; **p *<* 0.01 (Student’s *t* test). **g**, **h** SKBR3 and BT474 cells were treated with lapatinib (1 μM) and THZ1 (50 or 250 nM) for 24 h. mRNA expression levels were determined using Q RT-PCR. Data represent mean ± SD (*n* = 3). *p *<* 0.05; **p *<* 0.01 (Student’s *t* test)

Based on the hypersensitivity of HER2+ BC cells to THZ1, we next investigated whether HER2 expression affects CDK7 activity tumor cells. Ectopic expression of human wild-type HER2 increased protein levels of both CDK7 and RNA Pol II, and phosphorylation of RNA Pol II CTD in immortalized human mammary epithelial (HMEC) cells (Fig. 3a). Consistent with these findings, de-induction of HER2 expression in HER2+ mouse mammary tumors derived from *MMTV-rtTA-TetO HER2* transgenic mice [9] resulted in a dramatic loss of CDK7 expression and decreased phosphorylation of RNA Pol II CTD (Fig. 3b). Inhibition of HER2 activity by lapatinib, a dual HER2/EGFR kinase inhibitor, decreased both phosphorylation and expression of CDK7, and phosphorylation of RNA Pol II CTD in the HER2+ BC cell line SKBR3 (Fig. 3c). Taken together, these results suggest that HER2 might regulate the expression and activity of the CDK7/RNA Pol II and may, as a result, mediate CDK7-dependent RNA Pol II phosphorylation and transcriptional initiation.

Given the role of CDK7 in phosphorylation of the RNA Pol II CTD at active genes [19, 23, 27], we hypothesized that a critical set of HER2 regulated genes (regulons) may confer sensitivity to CDK7 inhibition in HER2+ cells. We therefore first compared changes in the transcriptomes of two HER2+ BC cell lines (SBKR3 and BT474) after treatment with the HER2/EGFR inhibitor lapatinib or the CDK7 inhibitor THZ1. Gene expression profiling indicated that 14–20% and 24–28% of the transcriptome was modulated after 6 h treatment with lapatinib or THZ1, respectively (Supplementary Fig. S3a, b and Table S1). We expected that the CDK7 inhibitor THZ1 would disrupt a significant portion of the gene expression that is inhibited by lapatinib. Indeed, THZ1 treatment led to a reduction in steady-state mRNA levels in these two breast cancer cell lines and affected 37.5% (377/1005) of the genes that were downregulated by lapatinib treatment (Supplementary Fig. S3c). We thus identified a subset of genes showing sensitivity to both HER2 and CDK7 inhibitors.

In parallel, we also analyzed how many HER2 regulons were perturbated by CDK7 inhibition. We compared the transcriptional changes in HMEC-HER2 cells, which ectopically express human HER2 in an HMEC cell background (Fig. 3a), with vector control HMEC-pBABE cells. We found that 2367 genes (FDR < 0.1, *p* < 0.05) were differently modulated by overexpression of HER2 in HMEC cells. Gene ontology analyses of these HER2 regulons showed significant enrichment in the pathways of cytokine–receptor interaction, cell adhesion molecules (CAMs) and NOD-like receptor signaling. (Supplementary Table S2 and Supplementary Fig. S4a). The 1274 genes were upregulated by HER2 overexpression in HEMC cells. 11.1% (141/1274) were inhibited by the CDK7 inhibitor THZ1 in HER2+ BC cell lines SKBR3 and BT474 (Fig. 3d and Supplementary Table S2). These HER2 regulons inhibited by THZ1 included a substantial number of signaling molecules and transcription factors (Fig. 3e). Overall 11.8% (151/1274) of these genes that were suppressed by lapatinib mainly function as enzyme regulator activity and ATPases (Fig. 3d and Supplementary Fig. S4b). Only 43 HER2 up-regulons were inhibited both THZ1 and lapatinib (Fig. 3d). In this subset of genes, we confirmed three genes with established roles in breast cancer, including *HES4* [28], *CCND1* [9], *IGFBP3* [29], with quantitative RT-PCR analyses. mRNA levels of these genes were increased in HMEC-HER2 cells, compared the vector control cell HMEC-pBABE, and decreased by treatment with both lapatinib and THZ1 in SKBR3 and BT474 cells (Fig. 3f–h). Thus, the 141-gene set in HER2+ cells may collectively represent a HER2-specific vulnerability, which mediated by CDK7 inhibition in breast cancers.

### CDK7/RNA Pol II activity regulated by RTKs and downstream signaling pathways confers resistance to HER2 inhibitors

**Fig. 4:**
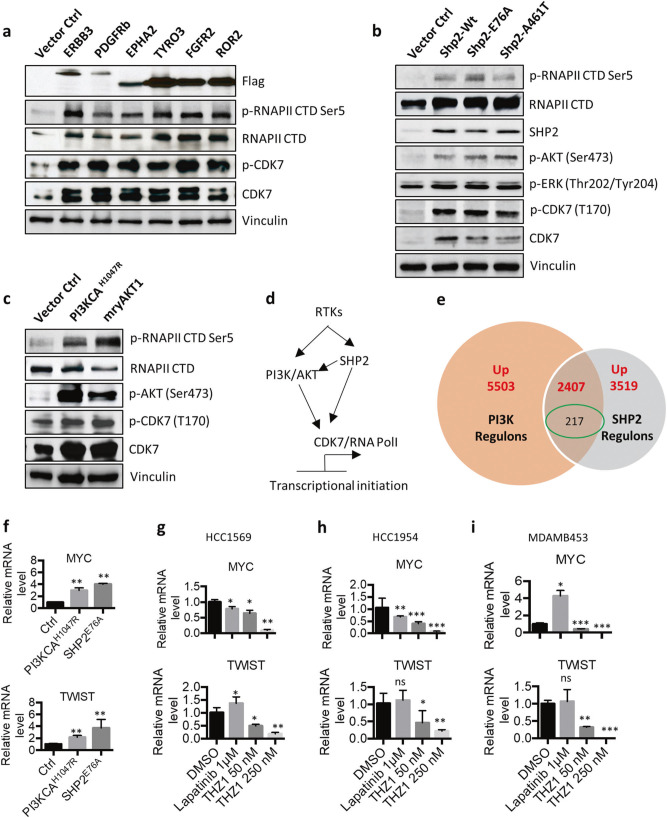
CDK7/RNA Pol II activity is regulated by receptor tyrosine kinases and their downstream pathways. **a** Expression and phosphorylation of CDK7 and RNA Pol II in HMEC cells stably overexpressing HER2 inhibitor-resistant RTKs. **b** Immunoblot analysis of ERK and AKT signaling pathways in HMECs with ectopic expression of wild-type and mutant SHP2. **c** Immunoblot analysis of CDK7 and RNA Pol II in HMECs with ectopic expression of mutant PI3KCA and wild-type AKT1. **d** Proposed mechanism of CKD7 transcriptional regulation by RTKs. **e** Overlap of genes that were upregulated by PI3K and SHP2 in HMECs. Genes inhibited by THZ1 in HER2+ BCs were circled in green. **f** Q RT-PCR analysis mRNA expression of MYC and TWIST in both HMEC-PIK3CA^H1047R^ and HEMC-SHP2^E76A^ HER2 cells, compared the vector control cell HMEC-pBABE, Data represent mean ± SD (*n* = 3). **p *<* 0.01 (Student’s *t* test). **g**, **h**, **i** Cells were treated with lapatinib or THZ1 for 24 h and the mRNA levels were determined using Q RT-PCR. Data represent mean ± SD (*n* = 3). *p *<* 0.05; **p *<* 0.01; ***p *<* 0.001 (Student’s *t* test)

One of the dominant mechanisms of intrinsic and acquired resistant to HER2-targeted therapies is the activation of compensatory signaling pathways. Multiple RTKs, including ERBB3, PDGFRB, EPHA2, TYRO3, FGFR2, and ROR2, have been reported to mediate the therapeutic resistance of HER2+ BC [30–32]. To explore whether these RTKs also promote the activity of CDK7, we established multiple cell models that stably overexpressed each HER2 inhibitor-resistant RTK (HER2iR RTK) using pWZL retroviral transduction in a HMEC cell line. All HER2iR RTKs tested induced the expression of both CDK7 and RNA Pol II and enhanced their activity by increasing phosphorylation levels (Fig. 4a).

The nonreceptor protein tyrosine phosphatase SHP2, which lies downstream of many RTKs, functions as an oncogenic tyrosine phosphatase. Inhibition of SHP2 selectively inhibits cancers driven by RTKs [33], suggesting that SHP2 serves as a central node downstream of RTK signaling. We showed that the dual SHP2 inhibitor PHPS1 [34] effectively sensitized human HER2iR breast cancer cells to lapatinib (Supplementary Fig. S5a–d), suggesting that aberrant SHP2 signaling may confer resistance to HER2 inhibitors in HER2+ BC cells. Indeed, we recently discovered a novel mutant form of SHP2 (A465T) in mouse HER2iR mammary tumors by RNA-seq analysis [9]. Ectopic expression of human analogous SHP2 (A461T) increased RNA Pol II phosphorylation through CDK7 by activating both ERK and AKT signaling pathways in HMECs (Fig. 4b), similar to previous findings with the WT protein and active E76A mutant [35]. These results suggest that SHP2 (A461T) is an active mutant form of the SHP2 protein and activation of SHP2 confers the resistance to HER2 inhibition in HER2+ BC cells.

Activation of PI3K signaling downstream of HER2 is one mechanism that may provide the progrowth and prosurvival signaling necessary to overcome HER2 inhibition in tumors. Expression of mutant PIK3CA or loss of PTEN in breast cancer cell lines is associated with resistance to HER2-targeted therapies [6, 36, 37]. We ectopically activated PI3K signaling by stably expressing oncogenic PI3KCA or AKT1 in HMEC cells. Overexpression of mutant PIKCA or wild-type AKT1 increased the phosphorylation and activity of RNA Pol II by increasing the expression of CDK7 (Fig. 4c). Taken together, our results suggest that the expression and activity of CDK7 is modulated by HER2iR RTKs and their downstream signaling kinases (Fig. 4d).

### CDK7 induces aberrant gene upregulation that confers resistance to HER2 inhibition

We further interrogated the role of CDK7 in the regulons of HER2iR kinases. We utilized our HEMC cell models and analyzed the alteration of the transcriptome driven by activation of PI3K or SHP2, two core intracellular kinases downstream of RTKs [33, 38]. We compared the transcriptomic changes of either HMEC-PIK3CA^H1047R^ or HMEC-SHP2^E76A^ to their vector control cells HMEC-pBABE. A total of 10908 genes and 7282 genes (FDR < 0.1, *p* < 0.05) were differentially expressed in HEMC-PIK3CA^H1047R^ cells or HEMC-SHP2^E76A^ cells, respectively, compared with HEMC-pBABE cells. Activation of PI3K by PIK3CA^H1047R^ enriched genes in the pathways of DNA replication, cellular senescence, cytokine–cytokine receptor interaction and PI3K-AKT signaling (Supplementary Fig. S6a). Activation of SHP2 regulated genes that were mainly involved in cytokine–cytokine receptor interaction and PI3K-AKT signaling pathway (Supplementary Fig. S6b), 67.8% (4936/7282) of SHP2 regulons are synchronously regulated by PI3K. Of these, 68.4% (2407/3519) of SHP2 up-regulons were upregulated by PI3K. These results further confirmed that PI3K is dominant downstream of SHP2 signaling in breast cells [35]. Nine percent (217/2407) PI3K/SHP2 up-regulons were inhibited by the CDK7 inhibitor THZ1 in HER2+ BC cell lines (Fig. 4e). These genes are enriched in transcriptional regulators, including TWIST and MYC, that are known to mediate HER2 inhibitor resistance [39, 40]. Quantitative RT-PCR analyses confirmed that mRNA levels of TWIST and MYC were increased in both HMEC-PIK3CA^H1047R^ and HEMC-SHP2^E76A^ HER2 cells, compared the vector control HMEC-pBABE cells. mRNA levels of these two genes were decreased by treatment with THZ1, but mostly not by lapatinib, in the HER2iR cell lines HCC1569, HCC1954, and MDAMB453 (Fig. 4f–i). Taken together, these results suggest that CDK7 mediates gene transcription engaged by multiple HER2iR kinase pathways. This subset of genes encoding transcriptional regulators and signaling factors in HER2-positive cells may partially represent a HER2iR vulnerability, which is mediated by CDK7 in breast cancers.

### Combined HER2-CDK7 inhibition synergistically suppresses tumor cell proliferation

Inhibition of CDK7 was reported to suppress an adaptive response to lapatinib via transcription repression in gastric and esophageal cancer cell models [41]. We asked if this also holds true in breast cancer cells. In HER2+ BC cells SKBR3 and BT474, THZ1 not only inhibited a set of genes that were downregulated by lapatinib (Supplementary Fig. S3c), but also suppressed the expression of a large number of genes that were upregulated by lapatinib. A total of 814 genes that were commonly upregulated by lapatinib in both SKBR3 and BT474 cells (Supplementary Table 1 and Supplementary Fig. S7a). Gene ontology terms showed that these genes were enriched in gene functioning in the regulation of transcription, kinase activity, and protein ubiquitination (Supplementary Fig. S7b), including SMAD2/3 signaling, FOXO signaling, and the HIF1-alpha transcription factor network (Supplementary Fig. S7c). Among these genes, 174 (21.3%) were inhibited by THZ1 in both cell lines, suggesting inhibition of CDK7 contributes to suppressing aberrant activation of transcriptome caused by lapatinib.

**Fig. 5:**
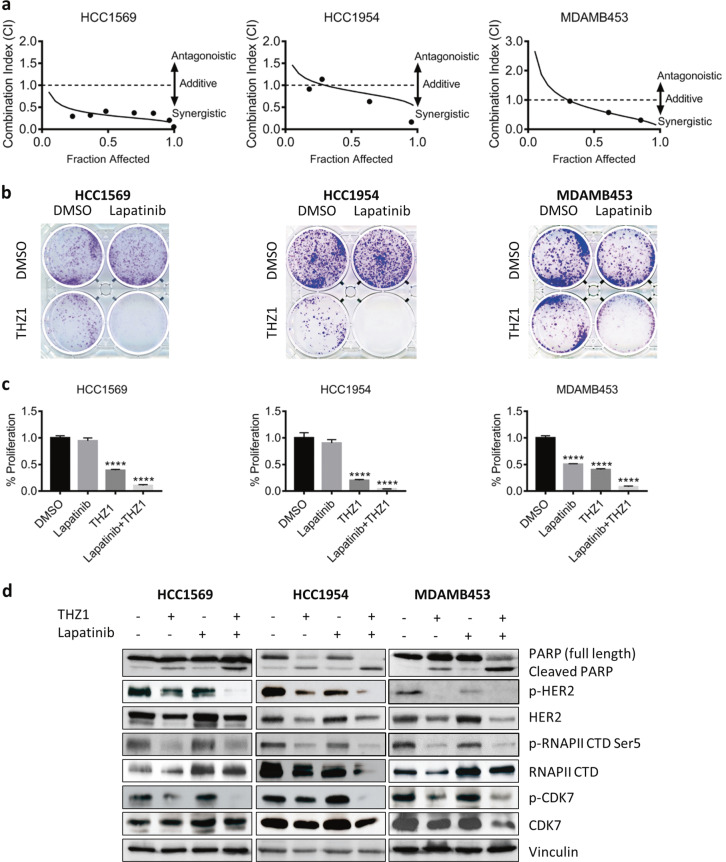
THZ1 in combination with lapatinib enhances cell death in HER2iR BC cells. **a** Combination index (CI) plots. Fraction affected refers to the proportion of cells with inhibition of viability. Dose-response curves of lapatinib combined with THZ1 were determined for each cell line. CI values were calculated using the median-effect equation described by the Chou–Talalay method. CI < 1 indicates synergism, CI = 1 indicates an additive effect, and CI > 1 indicates antagonism. **b**, **c** Effect of lapatinib and THZ1 on clonogenic potential. After treatment of breast cancer cells up to 2 weeks, colonies were stained with crystal violet and the number of colonies was quantified. Data represent mean ± SD of three replicates. ****p < 0.0001 (one-way ANOVA). **d** HCC1569, HCC1954, and MDAMB453 cells were treated with vehicle control (DMSO), THZ1 (100 nM) and lapatinib (1 μM) alone or in combination for 24 h before immunoblotting using the indicated antibodies

We then ask if inhibition of CDK7 resensitizes breast cancer cells to lapatinib, a HER2-targeted agent commonly used in patients with HER2+ metastatic breast cancer. Specifically, we tested whether inhibition of CDK7 restored the sensitivity of three HERiR tumor cell lines HCC1569, HCC1954, and MDAMB453, which are intrinsically resistant to HER2 blockade. We used the Chou–Talalay method for drug combination studies [42] and determined the dose-response curves of lapatinib and THZ1 individually and in combination at a constant potency ratio. For each of the cell lines, we observed the single-agent activity of lapatinib and THZ1 and a remarkably potent synergistic inhibition of cell viability after combined treatment. The calculated combination index (CI) values for HCC1569, HCC1954, and MDAMB453 cells at doses that reduced cell viability by 50% were 0.44, 0.74, and 0.52, respectively (Fig. 5a and Supplementary Fig. S8a–f).

To examine the long-term effects of HER2 and CDK7 inhibition, we determined the effects of lapatinib and THZ1 on clonogenic growth of these three cell lines. Cells were exposed to low doses of lapatinib (0.25 or 0.5 μM) and THZ1 (5 or 10 nM) individually or in combination for up to 2 weeks. Although treatment with THZ1 alone suppressed clonogenic growth, combined THZ1 and lapatinib treatment were more effective (Fig. 5b). Quantitation of colonies revealed a greater reduction in the number of colonies after combined treatment with THZ1 and lapatinib than with the individual inhibitors (Fig. 5c).

We next assessed the activities of CDK7/RNA Pol II complex in response to lapatinib and THZ1 alone or in combination in these three cell lines. Unlike in HER2iS cell line SKBR3 (Fig. 3c), lapatinib did not obviously suppressed the expression and phosphorylation of CDK7 in all three HER2iR cell lines. THZ1 not only suppressed phosphorylation of RNA Pol II, but also disrupted mRNA and protein levels of HER2 (Fig. 5d, Supplementary Fig. S9a, b), subsequently resulting in reduced phosphorylated HER2 in these cells (Fig. 5d and Supplementary Fig. S9c). Combined treatment with CDK7 and lapatinib greatly inhibited both HER2 activation and RNA Pol II phosphorylation and enhanced tumor cell apoptosis as assessed by increased levels of the active form of PARP (Fig. 5d). Therefore, CDK7 inhibition restores sensitivity of HER2iR tumor cells to the effects of HER2 inhibitors through decreased cell proliferation and increased induction of apoptotic cell death.

### The effects of combined CDK7 and HER2 inhibition on tumors in vivo

**Fig. 6:**
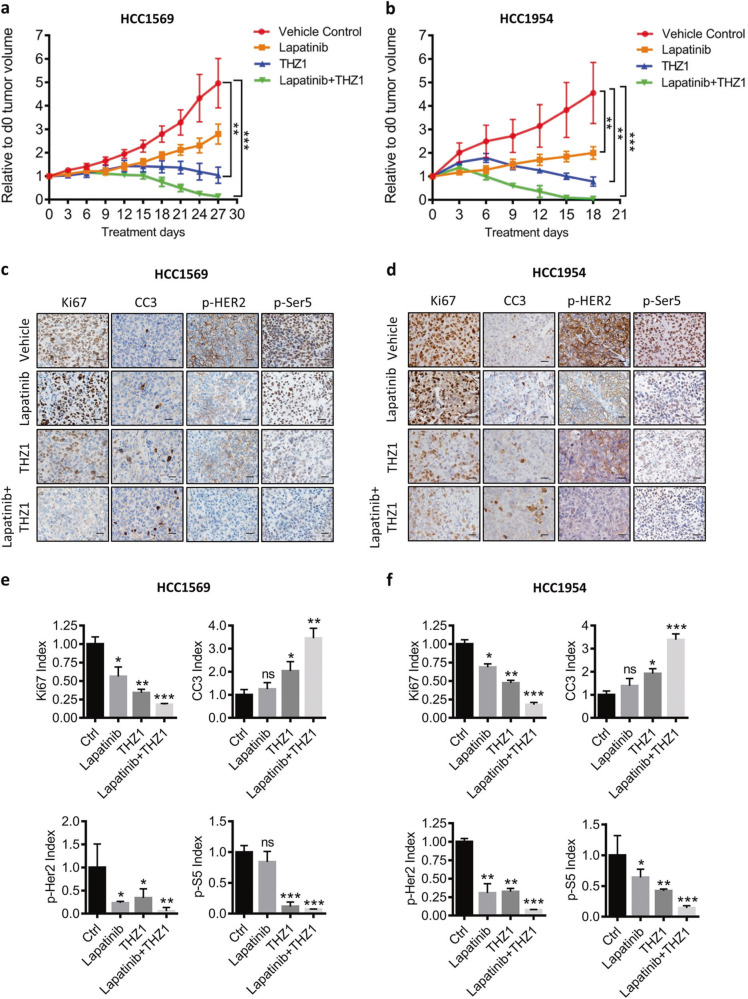
Combined treatment with lapatinib and THZ1 impairs in vivo cell growth in HER2iR breast cancer xenografts. **a**, **b** Growth of HCC1569 or HCC1954 tumors in nude mice treated with vehicle, lapatinib (100 mg/kg) and THZ1 (10 mg/kg) alone, or in combination for the indicated time. Values represent mean ± SEM. **p *<* 0.01; ***p *<* 0.001 (one-way ANOVA). **c**, **d** Immunohistochemical analysis of proliferation (Ki67), apoptosis cleaved caspase 3 (CC3), phospho-HER2, and phospho-S5 levels in harvested tumors. Scale bar represents 20 μm. **e**, **f** Quantitative analyses of three randomly selected IHC images per tumor sections (Tn = 6). Data represent mean ± SEM. *p *<* 0.05; **p *<* 0.01; ***p *<* 0.001 (Student’s *t* test)

Last, we tested if CDK7 could resensitize HER2iR breast cancer cells to lapatinib in vivo. NCI nude mice bearing palpable HCC1569 or HCC1954 xenografts were treated with lapatinib and THZ1 alone or in combination. Lapatinib alone resulted in marginal inhibition of tumor growth, whereas THZ1 alone significantly impaired tumor growth (Fig. 6a, b and Supplementary Fig. S10a, b). Combined HER2/CDK7 inhibition resulted in durable tumor regression in the HCC1569 and HCC1954 xenograft tumor models. Together, these data suggest that although CDK7 is a good target to overcome resistance to HER2 therapy in HER2+ BCs, combined inhibition of HER2 and CDK7 offers maximal cell growth inhibition and durable tumor regression.

To evaluate signaling and pharmacodynamic responses of HCC1569 and HCC1954 xenografts during inhibitor treatment we isolated tumors 72 h after drug administration and analyzed molecular markers. Dual HER2 and CDK7 inhibition decreased RNA Pol II phosphorylation, suppressed cell proliferation, and induced apoptosis (Fig. 6c–f). These results establish that CDK7 primarily exerts its transcriptional kinase activity by mediating signaling through HER2 and other RTKs in HER2+ breast tumors.

## Discussion

Here we show that a CDK7 inhibitor, THZ1, strongly inhibited HER2+ BC cell growth and increased apoptosis, even in cancer cells that exhibited resistance to HER2-targeted therapy. Unexpectedly, CDK7 did not promote cell cycle progression directly through phosphorylation of CDKs and ER, indicating that CDK7 plays its primary role in transcription in these cells. Using HMEC cell models, we demonstrated that the transcriptional activity of CDK7 was modulated by HER2, other RTKs and their downstream PI3K and SHP2 signaling nodes. Our studies also demonstrated a novel codependency of HER2 and CDK7 that might be exploited in therapeutically recalcitrant HER2+ BC. Our results also suggest that patients with HER2+ BC may have a durable response to combined inhibition of HER2 and CDK7.

Multiple kinases can contribute to escape from lapatinib-mediated growth inhibition, consistent with a shift in dependency to alternative signaling nodes in addition to HER2. This prevents their targeting by a single kinase inhibitor and underscores the difficulty in choosing the most effective kinase inhibitor combinations to treat HER2+ tumors. We approached this problem with the hypothesis that lapatinib would have a more durable effect in inhibiting HER2+ cell growth if we could block the signaling of multiple HER2iR kinases. We therefore targeted proteins involved in transcriptional regulation of RTKs that drive the signaling networks responsible for lapatinib resistance. CDK7 functions as a core transcriptional kinase that mediates the activity of multiple RTKs capable of generating resistance to HER2-targeted therapies. By inhibiting signaling of multiple RTKs through CDK7 inhibition we could achieve durable growth inhibition and potentially block the adaptive responses seen to a single RTK inhibitor.

TNBC and ER+ BC cells have been reported to respond to CDK7 inhibition through different mechanisms [20, 22]. TNBC cells depend on the transcriptional role of CDK7 and possess a cluster of TNBC-specific genes that are especially sensitive to CDK7 inhibition, whereas in ER+ cells THZ1 blocks phosphorylation of mutant ER at Ser118 and inhibits cell growth. We analyzed EGFR, another HER family member frequently overexpressed in TNBCs, and found that, similar to HER2 case, there was activation of the CDK7/RNA pol II complex by two active mutants, EGFR^L858R^ [43] and EGFR^Del^ [43], in our HEMC cell system (Supplementary Fig. S11), suggesting that EGFR signaling drives CDK7 mediated transcription in TNBCs.

Our finding that both HER2 inhibitor-sensitive and -resistant breast cancer cell lines became highly sensitized to THZ1 and exhibited a similar response to THZ1 to that of TNBC, could be attributed to CDK7-dependent transcriptional inhibition[7]. We examined the role of CDK7 in gene transcription, cell cycle activation, and ER phosphorylation in two HER2+/ER+ cell lines (BT474 and MDAMB453), showed that CDK7 primarily functions in gene transcription. It is worth noting that the cell cytotoxicity induced by CDK7 inhibition affects the growth of HER2+ BC cells in a HER2-dependent and -independent manner. In HER2iS cells, CDK7 mainly mediates gene transcription activated by HER2 signaling (Fig. 3d), whereas in HER2iR cells CDK7 plays a critical role in halting gene transcription activated by intrinsic and acquired signaling through multiple dominant HERiR kinases (Fig. 5d). We extended our study to other cancer models with a second kinase driven lineage, which exhibited resistance to a primary targeted kinase inhibitor. We tested the sensitivity of lung cancer cell lines PC9 and PC9GR4 to THZ1. PC9 cells are known to contain a deletion in exon 19 (DelE746A750) of EGFR, while PC9GR4 cells were obtained by chronic exposure to the EGFR inhibitor gefitinib and feature both activation of the FGF2-FGFR1 signaling pathway, as well as harboring an additional EGFR mutant, T790M, that is resistant to gefitinib [44]. Both PC9 and PC9GR4 exhibited a high sensitivity to THZ1 with low IC50 values (Supplementary Fig. S12a). We also examined a melanoma cell line A375^MEK1Q56P^, which exhibits resistance to BRAF inhibitors [45]. A375^MEK1Q56P^ showed less response to THZ1 treatment compared with the parental cell A375 with BRAF^V600E^ (Supplementary Fig. S12b). These results suggest inhibition of CDK7 may serve as an additional therapeutic avenue that prevents reactivation of multiple RTKs, but is not capable of silencing all the nodes of downstream intracellular signaling. Further experimentation in this area will be required.

Aberrant activation of SHP2 has been implicated in several human diseases and malignancies [46]. Although somatic SHP2 mutations are rare in solid tumors [47], aberrant activation of upstream RTKs, such as the overexpression of HER2 in breast cancer [48] and EGFR in colon cancer [49], has been shown to activate SHP2 to promote cancer development, progression, and resistance to targeted therapies. Here, we provide the first evidence that aberrant activation of SHP2 mediates resistance of breast cancer cells to HER2 blockade and this resistance can be overcome by inhibition of CDK7. In HER2+ BC, both PI3K and MAPK can mediate resistance of cancer cells to HER2-targeted therapy [7, 9]. PI3K and MAPK signaling pathways are dominantly active in TNBC; nearly 80% of TNBCs have potential MAPK/ERK pathway activation and 90% exhibit PI3KCA pathway activation [50]. Thus, it is not surprising that 15% (9/60) of the CDK7-sensitive Achilles cluster of genes in TNBCs [20] are also inhibited by THZ1 in HER2+ BC cells (SKBR3 and BT474) (Supplementary Table 1). To explore the role of SHP2, PI3K, and ERK signaling in the resensitization of HER2iR cells to lapatinib by the addition of THZ1, we examined the activity and expression of SHP2, and downstream PI3K/AKT and ERK signaling by western blotting in three HER2iR cell lines HCC1569, HCC1954, and MDAMB453. PI3K and ERK signaling, independent of SHP2, played roles in the two cell lines HCC1569 and HCC1954 treated by THZ1, while, noncanonical signaling downstream of SHP2, rather than PI3K and ERK signaling, appears to mediate resensitization to lapatinib by THZ1 in MDAMB453 cells. These results suggest that specific dominant HER2iR RTKs or their downstream signaling nodes control the activity of CDK7/RNA pol II complex and drive CDK7 mediated transcription activation in each individual HER2iR breast cancer cell line (Fig. 5d and Supplementary Fig. S13). The HER2 regulon cyclin D1 is chiefly regulated via both the MAPK/ERK and PI3K pathways[51, 52]. We recently reported that a high level of cyclin D1 confers resistance to HER2 pathway blockade [9]. Here, we show that inhibition of CDK7 efficiently decreases the mRNA levels of *CCND1* in HE2iR breast cancer cells (Supplementary Fig. S14). Taken together, these data suggest that CDK7 regulates HERiR-related transcriptomic changes activated by multiple kinase signaling pathways.

In summary, our data identify CDK7 as a new therapeutic target to improve the treatment and survival of patients with HER2+ BC and reveal how CDK7 acts as a transcriptional kinase in these cancers. This study provides the first demonstration that targeting CDK7-dependent transcription is an effective method to globally suppress the multiple kinase-activated genes that are critical for proliferation and viability of HER2+ BC cells. We show that CDK7 inhibition decreases transcriptional levels of multiple genes engaged by RTKs and downstream PI3K/AKT and SHP2 signaling. Combined inhibition of HER2 and CDK7 maximally abrogated RNA Pol II activation and induced a durable tumor response. Our findings reveal a novel codependency of HER2 and CDK7 that can be exploited in therapeutically recalcitrant HER2+ BC.

## Materials and methods

The following methods are detailed in Supplementary Information, including the source of cell culture and reagents, transfection of siRNA and plasmids, cell viability assay, cell cycle analysis and annexin V staining, western blotting, Gene expression profiling by RNA sequencing (RNA-seq) and microarray, immunofluorescence, quantitative real-time PCR, mouse xenotransplantation experiments, histology and immunohistochemistry (IHC), and data statistical analyses.

### Supplementary information

Supplementary Information

Supplementary Table 1

Supplementary Table 2
