# Correction: Inhibition of the transcriptional kinase CDK7 overcomes therapeutic resistance in HER2-positive breast cancers

**DOI:** 10.1038/s41388-024-02999-1

**Published:** 2024-05-29

**Authors:** Bowen Sun, Seth Mason, Robert C. Wilson, Starr E. Hazard, Yubao Wang, Rong Fang, Qiwei Wang, Elizabeth S. Yeh, Meixiang Yang, Thomas M. Roberts, Jean J. Zhao, Qi Wang

**Affiliations:** 1 The First Affiliated Hospital, Biomedical Translational Research Institute and School of Pharmacy, Jinan University, Guangzhou, 510632 China; 2 Department of Pathology and Laboratory Medicine, Medical University of South Carolina, Charleston, SC 29425 USA; 3 Computational Biology Resource Center, Medical University of South Carolina, Charleston, SC 29425 USA; 4 Department of Cancer Biology, Dana-Farber Cancer Institute, Boston, MA 02115 USA; 5 Department of Biological Chemistry and Molecular Pharmacology, Harvard Medical School, Boston, MA 02115 USA; 6 Department of Pathology, Zhejiang Provincial Key Laboratory of Pathophysiology, Ningbo University School of Medicine, Ningbo, 315211 China; 7 Department of Cell and Molecular Pharmacology and Experimental Therapeutics, Medical University of South Carolina, Charleston, SC 29425 USA

Correction to: *Oncogene* 10.1038/s41388-019-0953-9, published online 28 August 2019

Following publication of this article, an error was noted in Fig. 1b with the same image being published for BT474 THZ1 and HCC1569 THZ1. After assessing the raw data, the authors confirmed the published image was correct for HCC1569 THZ1, but not BT474 THZ1. The correct image for BT474 THZ1 is provided below.
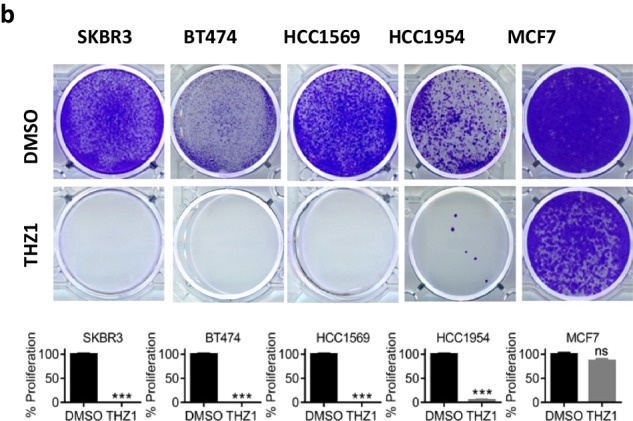


The authors confirm this correction has no impact on the results and apologise for any inconvenience caused by the error.

The original article has been corrected.

