# Supplementary material for: Inhibition of the transcriptional kinase CDK7 overcomes therapeutic resistance in HER2-positive breast cancers
Source: Oncogene. 2019 Aug 28;39(1):50–63. doi: 10.1038/s41388-019-0953-9 (PMC6937212; doi:10.1038/s41388-019-0953-9)
Supplement: Supplementary file 1 — Supplementary Information [file 41388_2019_953_MOESM1_ESM.pdf]

Inhibition of the transcriptional kinases CDK7 overcomes therapeutic resistance in HER2-positive breast cancers.

Bowen Sun[1,2], Seth Mason[2], Robert C. Wilson[2], Starr E. Hazard[3], Yubao Wang[4,5], Rong Fang[4,5,6], Qiwei Wang [4,5], Elizabeth S. Yeh[7], Meixiang Yang[1], Thomas Roberts[4,5], Jean J. Zhao[4,5], and Qi Wang[2]

Supplementary materials and methods

Cell culture and reagents

Breast cancer cell lines MDAMB361, MDAMB53, MDAMB468, SKBR3, HCC1954, CAMA-1, MCF7, T47D and lung cancer cell lines PC9, PC9GR4 were generously provided by the laboratory of Jean Zhao at the Dana Farber Cancer Institute. Sum149 was obtained from Stephen Ethier's laboratory at the Medical University of South Carolina. HCC1569, BT474, BT483 and malignant melanoma cell lines A375, and A375^{MEK1Q56P} were purchased from the American Type Culture Collection (ATCC, Manassas, VA, USA). MDAMB361, MDAMB453, MDAMB468 A375 and A375^{MEK1Q56P} were cultured in DMEM (GIBCO, NY, USA) supplemented with 10% FBS (Corning, NY, USA) and 1% penicillin/streptomycin (GIBCO). SKBR3, BT474, HCC1569, HCC1954, T47D, PC9 and PC9GR4 were cultured in RPMI-1640 (GIBCO) supplemented with 10% FBS and 1% penicillin/streptomycin. CAMA-1 was cultured in MEM (GIBCO) supplemented with 10% FBS and 1% penicillin/streptomycin. BT483 was cultured in RPMI-1640 supplemented with 20% FBS, 1% penicillin/streptomycin, and 0.01 mg/ml insulin (Sigma-Aldrich, St. Louis, MO, USA) solution from bovine pancreas (Sigma).

Sum149 was cultured in Ham's F-12 medium (GIBCO) supplemented with 5% FBS, 1% penicillin/streptomycin, 5 µg/ml insulin (Invitrogen, CA, USA), and 1 mg/ml hydrocortisone (Sigma).

Pre-malignant cell models HMEC-HER2, HMEC-ERBB3, HMEC-PDGFRb, HMEC-EPHA2, HMEC-TYRO3, HMEC-FGFR2, HMEC-ROR2, HMEC-P13KCA^{H1047R}, HMEC-mryAKT1, HEMC-EGFR^{L858R} and HMED-EGFR^{del} were generated by pWZL or pBABE retroviral introduction. These cells were cultured in DMEM/F12 (Invitrogen) supplemented with 3 ml FBS with cholera toxin (in 500 ml medium), 1% penicillin/streptomycin, 1% Antibiotic-Antimycycotic (GIBCO), 10 ng/ml epidermal growth factor (human) (EGF) (Sigma), 500 ng/ml hydrocortisone, and 10 µg/ml insulin[12].

SHP2 wild-type and mutant vectors pBABE-SHP2 WT and SHP2 E76A were purchased from Addgene (Watertown, MA, USA). The Quick change lighting multi-site mutagenesis kit (Agilent) was used to generate the SHP2 A461T mutation with the following mutagenesis primers: A461T forward: 5´-GTG GTG CAC TGC AGT ACT GGA ATT GGC CGG A-3' and reverse, 5´-TCC GGC CAA TTC Cag TAC TGC AGT GCA CCA C-3´. Retroviruses produced by 293 phoenix cells were used for infection of HMEC cells in media containing polybrene (8 µg/ml). After infection, cells were selected with puromycin and cultured in HMEC medium.

Chemicals

Lapatinib ditosylate (HY-50898A), THZ1 hydrochloride (HY-80013A) were purchased from MedChem Express (Monmouth Junction, NJ, USA). Triptolide (3253) and Purvalanol A

(1580) were purchased from Tocris Bioscience (Missouri, USA). PHPS1 sodium salt hydrate (P0039) was purchased from Sigma Alcrich.

Cell viability assay

Cells were seeded in 96-well plates at a density of 6 x 10^3 cells/well and treated with THZ1 (ranging from 1 nM to 10 μM) for 72 h. Cell viability was assessed using the CellTiter-Glo Luminescent Cell Viability Assay (Promega, Madison, WI, USA). The long-term cologenic growth was follow the protocol described previously[8] In brief, cells were seeded in 6-well plates at a density of 8000 cells/well for 24 hours, and indicated concentrations of inhibitors were added. After 12-14 days, cells were fixed in 2.5% buffered formalin and stained in 0.1% (w/v) crystal violet solution for 15 minutes each. The number of colonies was quantified by Image-Pro Plus system.

Isobologram and combination index analysis

Cells were seeded in 96-well plates at a density of 6 x 10^3 cells/well and treated with Lapatinib (ranging from 0.0625 μM to 20 μM), THZ1 (ranging from 6.25 nM to 400 nM) or combination for 72 h. The concentrations used corresponded to 0.0625, 0.125, 0.25, 0.5, 1, 2, 4 times the IC50 of each agent. The concentration of the single drug that inhibits 50% of cell proliferation (IC50) was determined by fitting the dose-response curve utilizing the CompuSyn software. To calculate the Combination Index (CI), using the method of constant ratio drug combination proposed by Chou and Talalay and described in result. The CI was calculated using the formula: $C.I. = C_{A,\,X}/IC_{X,\,A}+C_{B,\,X}/IC_{X,\,B}$, where $C_{A,\,X}$ and $C_{B,\,X}$ are the concentration of lapatinib and THZ1 used in combination to achieve 50% drug effect. $IC_{X,\,A}$

and $IC_{X, B}$ are the concentrations for single agents to achieve the same effect. A CI of less then, equal to, or more than 1 indicates synergic, additive or antagonistic effect, respectively.

Cell cycle analysis and Annexin V staining

After THZ1 treatment, culture supernatant and trypsinized cells were combined and centrifuged. After two washes in cold PBS, cells were fixed with 80% ethanol overnight at 4ºC and resuspended in 1 ml PBS supplemented with 20 µl propidium iodine (PI) (BD Biosciences, San Jose, CA) (stock, 0.5 mg/ml in H_2O at 4ºC) and 10 µl RNase A (stock, 1 mg/ml) (Sigma). After incubation for 20 min at 37ºC in the dark, analysis was performed on a FACS AriaII cytometer (BD Biosciences) and cell cycle profiles were plotted as histograms. Apoptosis was analyzed using a FITC Annexin V/PI Apoptosis Detection Kit (BD Biosciences) according to the manufacturer's protocol. Annexin V/PI assessments were generated using FlowJo software (Tree Star, FlowJo, Ashland, OR). Duplicate cell cultures for each treatment were analyzed in each experiment.

Western blotting

Cells were lysed in 1% NP-40 buffer supplemented with protease and phosphatase inhibitors (Roche, Basel, Switzerland, USA). Equal amounts of proteins were resolved by SDS-PAGE and transferred to nitrocellulose membranes for immunoblot analysis. Primary antibodies to the following proteins were used for immunoblotting: RNAPII CTD S2 (Bethyl laboratories inc, Montgomery, TX, USA A300-654A), RNAPII CTD S5 (Bethyl A304-408A), RNAPII CTD S7 (Millipore, Charlottesville, VA, USA 04-1570), RNA Pol II (Bethyl A300-653A), cleaved PARP (Cell Signaling Technology [CST], Danvers, MA, USA 9541), phospho-Rb (ser780) (CST

9307), Rb (CST 9309), E2F-1 (CST 3742), phospho-cdc2 (Thr161) (CST 9114), CDK1/CDK2 (Santa Cruz Biotechnology sc-53219), phospho-CDK2 (T160) (CST 2561), CDK2 (78B2) (CST 2546), phospho-CDK7 (Abcam, Cambridge, MA, USA ab59987), CDK7 (Abcam ab137716), phospho-SHP2 (Y543) (Abcam 62322), SHP2 (phospho Y542) (Abcam ab62322), phospho-HER2 (CST 2243), HER2/ErbB2 (29D8) (CST 2165), phosphor-AKT (Ser473) (CST 4060), phospho-ERK1/2 (Thr202/Tyr204) (CST 9101), ERK1/2 (137F5) (CST 4695), Anti-Flag M2 (Sigma F1804), phospho-ERα (Ser118) (CST 2511), ERα (2Q418) (Santa Cruz Biotechnology sc-71064), EGF Receptor (D38B1) XP (CST 4267), and Vinculin (Sigma 12M4787V). After incubation with appropriate horseradish peroxidase (HRP)-linked secondary anti-mouse, anti-rabbit or anti-rat secondary antibodies (Bio-Rad, CA, USA) for 1 h at room temperature, the membrane was incubated with Enhanced Chemiluminescence Plus substrate (Pierce, USA) and signals were detected using Pierce CL-Xposure films.

Gene expression profiling by RNA sequencing (RNA-seq) and microarray

The transcriptome of HMEC-HER2, HMEC-SHP2^{E76A}, HMEC-PIK3CA^{H1047R} or HEMC^{-pBABE} cells was analyzed by RNA sequencing. Total RNAs from three independent cultures of each cell line were prepared using Qiagen RNeasy kits with on-column DNase digestion according to the manufacturer’s instructions. 100 ng of total RNA was used to prepare RNA-seq libraries using the TruSeq v2 RNA Sample Prep Kit following the manufacturer’s protocol (Illumina, San Diego, CA). RNA-seq libraries were paired-end sequenced with 2x125 cycles on a HiSeq 2500 (Illumina, San Diego, CA) with a minimum depth of 100 million reads. Four samples per lane were clustered on a cBot as described by the manufacturer. Clustered RNA-seq libraries were paired-end sequenced with 2x125 cycles on a HiSeq 2500.

Demultiplexing was performed utilizing CASAVA to generate Fastq files. We implemented stringent quality control of the Fastq files using FastQC. Then, reads were aligned to the gene annotations of UCSC hg19 human reference genome assembly using TopHat2[5] and STAR[2]. Aligned reads were converted from BAM to SAM format using SAMtools. The resulting SAM files were inputted into the Python package HTSeq and quantitative readouts for each sample obtained in the form of count data. The data (GSE127876) were assessable at: https://www.ncbi.nlm.nih.gov/geo/query/acc.cgi?acc=GSE127876. In order to infer differential signal within the data sets with robust statistical power, we utilized both DESeq2 and edgeR[6, 9], both of which tests for differential expression based on a model using negative binomial distribution. Transcript count data from DESeq2 analysis were ranked according to q-value, the smallest false discovery rate (FDR) at which a transcript is called significant.

Breast cancer cell liens SKBR3 and BT474 were treated with vehicle control (DMSO), THZ1 (250 nM) or lapatinib (1 μM) for 6 h. Total RNA (300 ng) from three independent cultures of each cell line was used to produce cRNA following the Illumina® Total PrepTM RNA Amplification Kit protocol (Illumina, San Diego, CA, USA). Amplified cRNA (750 μg) was hybridized to HumanHT-12 v4.0 Expression BeadChip arrays as described in the WGGEX Direct Hybridization Assay Guide (Illumina). Processed arrays were imaged with the HiScan array imager (Illumina). Array data were imported into Illumina's Genome Studio. The array was then exported for analysis using Partek's Genomics Suite (Partek Inc., Santa Clara, CA, USA). The data (GSE129254) were assessable at https://www.ncbi.nlm.nih.gov/geo/query/acc.cgi?acc=GSE129254. The statistical differential gene (GE) lists (FDR <0.1, p<0.05) from the RNA-seq and microarray experiments were

sorted since both experiments were reported to be consistent in analyzing perturbations in gene expression[3, 4]. Statistical analysis of pathways and gene ontology terms were will be carried out using the sorted transcript list, which is subjected to the Gene Set Enrichment Analysis and the ToppGene Suite[1, 7].

Quantitative real-time PCR

Total RNA was extracted with TRIzol reagent (Invitrogen) and 1-5 µg was used for synthesis of first-strand cDNA with Maxima First Strand cDNA Synthesis Kit (Thermo Fisher Scientific). Quantitative PCR was performed with Maxima SYBR Green qPCR Master Mix (Applied Biosystems, USA). The primer sequences used were as follows: GAPDH (5'-CCTGTTCGACAGTCAGCCG-3', 5'-CGACCAAATCCGTTGACTCC-3'). HES4 (qHsaCED0047964), CCND1 (qHsaCID0013833), IGFBP3 (qHsaCID0010824), MYC (qHsaCID0028650), TWIST (qHsaCID0014204) and HER2 (qHsaCEP0052301) were purchased from Bio-Rad. Real-time PCR was performed on a QuantStudio 6 Flex Real-Time PCR System (Applied Biosystems).

Xenograft studies

All animal experiments were conducted in accordance with animal use guidelines from the National Institutes of Health using protocols approved by the Medical University of South Carolina. To isolate mammary tumor from *MMTV-rtTA-TetO HER2* mice, Doxcycline induction was performed by introduction of a 2500-ppm doxycycline-containing diet. Mice were monitored twice weekly for the development of mammary tumors and tumors were measured by caliper twice weekly. To de-induce transgene expression, mice were switched

to a standard diet until tumor size reaches to 1.5 cm in diameter. Portions of the tumor tissues were isolated before and 96 hours after doxycycline withdrawal.

Breast cancer cells HCC1569 and HCC1954 were harvested and resuspended in 40% Matrigel-Basement Membrane Matrix, LDEV-free (BD Biosciences) and injected (100 μl per site) into the fourth pair of mammary fat pads of nude mice (CrTac: NCr-Foxn1nu). When palpable tumors had formed (~100 mm), the mice were randomly assigned into study groups (n=6) as follows: treatment with vehicle; lapatinib alone (daily by gavage, 100 mg/kg[10]); THZ1 alone (daily by intraperitoneal injection, 10 mg/kg[11]); and combination treatment with lapatinib and THZ1. Every three days tumor size was measured in two dimensions using manual calipers and tumor volume was calculated using the formula $V = 0.5 \times \text{length} \times \text{width}^2$. Upon harvest the tumors were fixed in formalin overnight and fixed in 70% ethanol for histopathology analysis.

Histology and immunohistochemistry (IHC)

Formalin-fixed paraffin-embedded sections were stained with hematoxylin and eosin in the Tissue Biorepository at Medical University of South Carolina. Immunohistochemical staining was performed with antibodies against Ki67 (Vector lab VP-K451), apoptosis cleaved-caspase 3 (CC3) (CST 9664), phospho-HER2 (CST 2243), and RNAPII CTD S5 (Bethyl A304-408A). For quantification of IHC, three randomly selected images were taken per tumor section and analyzed using Image-Pro Plus System.

Statistical Analyses

Values are expressed as means ± SEM or SD. Statistical evaluation was performed with Student's *t* test for paired data or ANOVA followed by Fischer's protected least significant difference test as appropriate. Values of $p < 0.05$ were considered to indicate a statistically significant difference.

Reference

1 Chen J, Bardes EE, Aronow BJ, Jegga AG. ToppGene Suite for gene list enrichment analysis and candidate gene prioritization. Nucleic acids research 2009; 37: W305-311.

2 Dobin A, Davis CA, Schlesinger F, Drenkow J, Zaleski C, Jha S *et al*. STAR: ultrafast universal RNA-seq aligner. Bioinformatics (Oxford, England) 2013; 29: 15-21.

3 Huff M, da Silveira WA, Carnevali O, Renaud L, Hardiman G. Systems Analysis of the Liver Transcriptome in Adult Male Zebrafish Exposed to the Plasticizer (2-Ethylhexyl) Phthalate (DEHP). Scientific reports 2018; 8: 2118.

4 Huff M, da Silveira W, Starr Hazard E, Courtney SM, Renaud L, Hardiman G. Systems analysis of the liver transcriptome in adult male zebrafish exposed to the non-ionic surfactant nonylphenol. General and comparative endocrinology 2019; 271: 1-14.

5 Kim D, Pertea G, Trapnell C, Pimentel H, Kelley R, Salzberg SL. TopHat2: accurate alignment of transcriptomes in the presence of insertions, deletions and gene fusions. Genome biology 2013; 14: R36.

6 Robinson MD, McCarthy DJ, Smyth GK. edgeR: a Bioconductor package for differential expression analysis of digital gene expression data. Bioinformatics (Oxford, England) 2010; 26: 139-140.

7 Subramanian A, Tamayo P, Mootha VK, Mukherjee S, Ebert BL, Gillette MA *et al*. Gene set enrichment analysis: a knowledge-based approach for interpreting genome-wide expression profiles. Proceedings of the National Academy of Sciences of the United States of America 2005; 102: 15545-15550.

8 Sun B, Jensen NR, Chung D, Yang M, LaRue AC, Cheung HW *et al*. Synergistic effects of SHP2 and PI3K pathway inhibitors in GAB2-overexpressing ovarian cancer. American journal of cancer research 2019; 9: 145-159.

9 Varet H, Brillet-Gueguen L, Coppee JY, Dillies MA. SARTools: A DESeq2- and EdgeR-Based R Pipeline for Comprehensive Differential Analysis of RNA-Seq Data. PloS one 2016; 11: e0157022.

10 Wang Q, Liu P, Spangle JM, Von T, Roberts TM, Lin NU *et al*. PI3K-p110alpha mediates resistance to HER2-targeted therapy in HER2+, PTEN-deficient breast cancers. Oncogene 2015.

11 Wang Y, Zhang T, Kwiatkowski N, Abraham BJ, Lee TI, Xie S *et al*. CDK7-dependent transcriptional addiction in triple-negative breast cancer. Cell 2015; 163: 174-186.

12 Zhao JJ, Gjoerup OV, Subramanian RR, Cheng Y, Chen W, Roberts TM *et al*. Human mammary epithelial cell transformation through the activation of phosphatidylinositol 3-kinase. Cancer cell 2003; 3: 483-495.

Supplementary figure legends

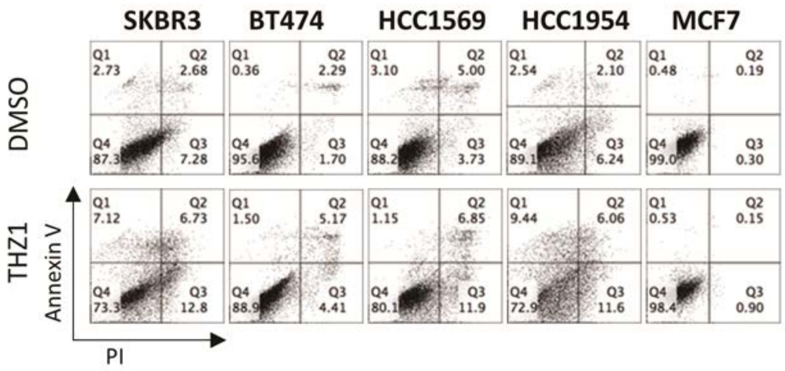

Supplementary Figure S1: Cell cycle progression in HER2+ breast cancer cells. Cell cycle analyses of cells after treatment with DMSO (vehicle control) or THZ1 (100 nM) for 24 h.

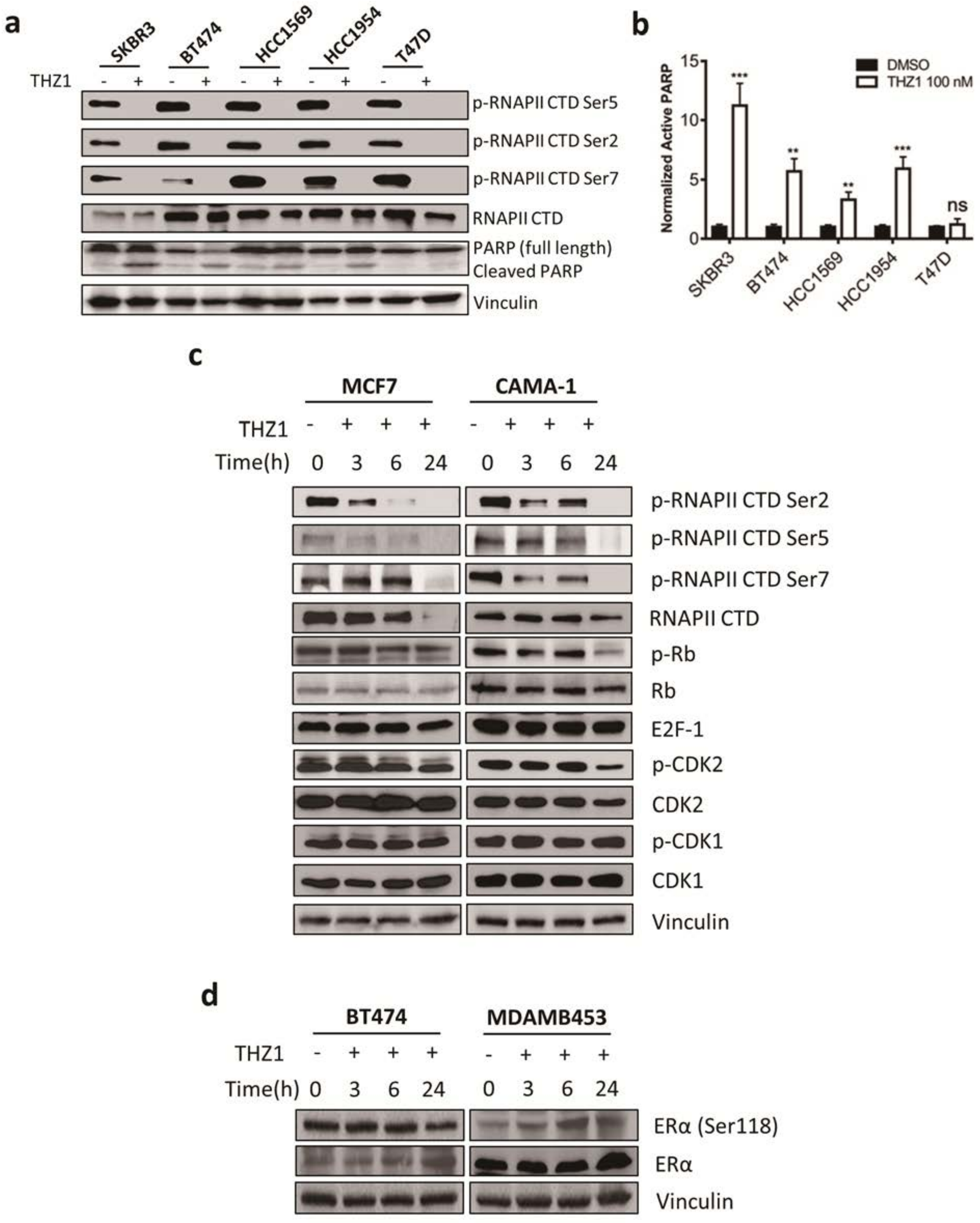
a
SKBR3
BT474
HCC1569
HCC1954
T47D
THZ1 - + - + - + - + - +
p-RNAPII CTD Ser5
p-RNAPII CTD Ser2
p-RNAPII CTD Ser7
RNAPII CTD
PARP (full length)
Cleaved PARP
Vinculin
b
Normalized Active PARP
DMSO
THZ1 100 nM
***
**
**
***
ns
SKBR3
BT474
HCC1569
HCC1954
T47D
c
MCF7
CAMA-1
THZ1 - + + + - + + +
Time(h) 0 3 6 24 0 3 6 24
p-RNAPII CTD Ser2
p-RNAPII CTD Ser5
p-RNAPII CTD Ser7
RNAPII CTD
p-Rb
Rb
E2F-1
p-CDK2
CDK2
p-CDK1
CDK1
Vinculin
d
BT474
MDAMB453
THZ1 - + + + - + + +
Time(h) 0 3 6 24 0 3 6 24
ERα (Ser118)
ERα
Vinculin

Supplementary Figure S2: Effects of CDK7 inhibition on phosphorylation in ER/PR and HER+/ER+ breast cancer cell lines. a, **b** Cells were treated with vehicle control (DMSO) or THZ1 (100 nM) for 0, 3, 6 and 24 h before immunoblotting using the indicated antibodies. Quantitation of PARP expression in each cell line with or without inhibitor treatment . Data represent mean ± SD from three independent experiments, *, $p < 0.05$; ns, not significant (Student's *t* test). **c** MCF7 and CAMA-1 cells were treated with vehicle control (DMSO) or THZ1 (100 nM) for 0, 3, 6 or 24 h before immunoblotting using the indicated antibodies. **d** BT474 and MDAMB453 cells were treated with vehicle control (DMSO) or THZ1 (100 nM) for 0, 3, 6 and 24 h before immunoblotting using the indicated antibodies.

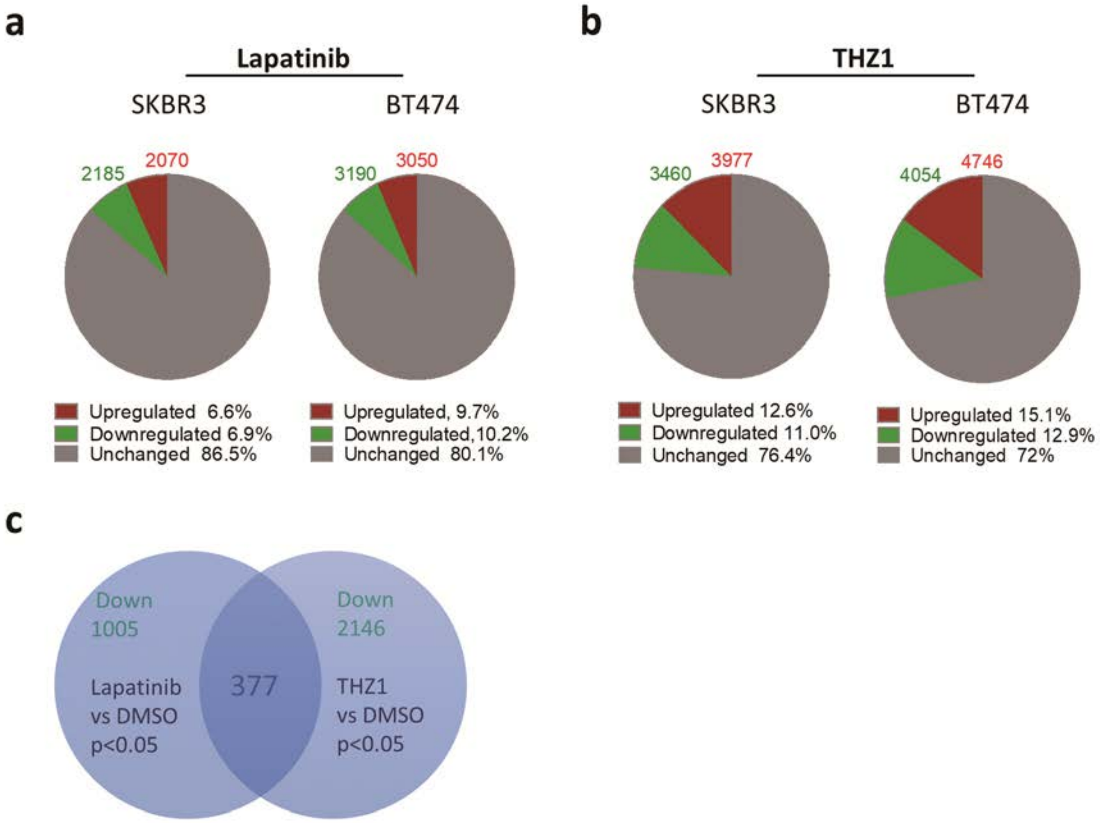

Supplementary Figure S3: a, **b Differentially expressed (DE) genes** in SKBR3 and BT474 cells after treatment with lapatinib (1 μM) or THZ1 (250 nM) for 6 h. Red, up-regulated genes; green, down-regulated genes. The application of multiple testing correction using benjamini and Hochberg false discovery rate (FDR). Data represent triplicates of each treatment group, $p < 0.05$. **c** Overlap between genes that were inhibited by (1 μM) or THZ1 (250 nM) for 6 h in both SKBR3 and BT474 cells.

a

Top pathways of differentially expressed genes driven by HER2

Pathway name	Pathway Id	p-value	p-value (FDR)	p-value (Bonferroni)
Cytokine-cytokine receptor interaction	04060	**8.131e-8**	**1.935e-5**	**1.935e-5**
Cell adhesion molecules (CAMs)*	04514	**1.924e-7**	**2.289e-5**	**4.579e-5**
Antigen processing and presentation	04612	**3.031e-7**	**2.404e-5**	**7.213e-5**
Influenza A	05164	**7.125e-7**	**4.239e-5**	**1.696e-4**
NOD-like receptor signaling pathway	04621	**1.711e-6**	**5.417e-5**	**4.072e-4**

* the p-value corresponding to the pathway was computed using only over-representation analysis.

b

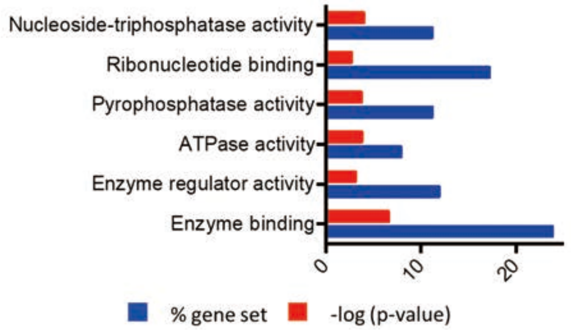

Supplementary Figure S4: Gene oncology analyses of HER2 up-regulons expression in HMEC cells. a Top pathways of HER2 regulons inhibited by THZ1 expression in HMEC cells. **b** Gene oncology analyses of HER2 up-regulons inhibited by lapatinib expression in HMEC cells.

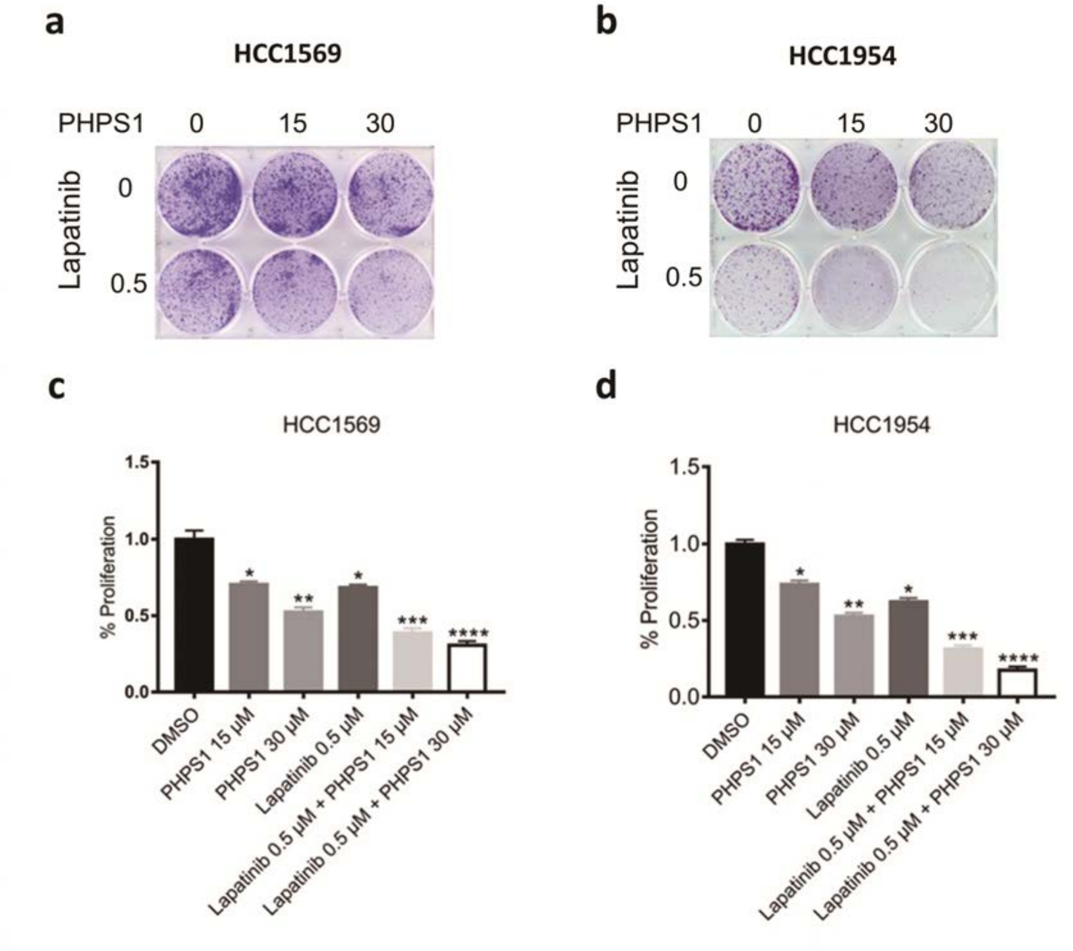

Supplementary Figure S5: SHP2 inhibitor PHPS1 effectively sensitized human HERiR breast cancer cells to lapatinib. HCC1569 and HCC1954 cells were treated with lapatinib with or without PHPS1 and vehicle control (DMSO) at the indicated concentrations. After 7-14 days the cell colonies were stained with crystal violet (**a**, **b**) and the number of colonies was quantified (**c**, **d**). Data represent mean ± SD of three replicates, *, $p < 0.05$; **, $p < 0.01$; ***, $p < 0.001$; ****, $p < 0.0001$ (one-way ANOVA).

a

Top pathways of differentially expressed genes driven by PI3KCA^{H1047R}

Pathway name	Pathway Id	p-value	p-value (FDR)	p-value (Bonferroni)
DNA replication*	03030	**1.199e-7**	**3.800e-5**	**3.800e-5**
Cellular senescence	04218	**3.190e-7**	**5.057e-5**	**1.011e-4**
Cytokine-cytokine receptor interaction	04060	**6.896e-7**	**7.287e-5**	**2.186e-4**
Pyrimidine metabolism*	00240	**4.224e-5**	**0.003**	**0.013**
PI3K-Akt signaling pathway	04151	**7.310e-5**	**0.005**	**0.023**

* the p-value corresponding to the pathway was computed using only over-representation analysis.

b

Top pathways of differentially expressed genes driven by SHP2^{E76A}

Pathway name	Pathway Id	p-value	p-value (FDR)	p-value (Bonferroni)
Cytokine-cytokine receptor interaction	04060	**1.120e-8**	**3.249e-6**	**3.249e-6**
PI3K-Akt signaling pathway	04151	**1.195e-7**	**1.733e-5**	**3.466e-5**
TNF signaling pathway	04668	**5.147e-7**	**4.975e-5**	**1.493e-4**
Complement and coagulation cascades	04610	**1.231e-6**	**8.925e-5**	**3.570e-4**
Amoebiasis	05146	**2.486e-6**	**1.442e-4**	**7.209e-4**

* the p-value corresponding to the pathway was computed using only over-representation analysis.

Supplementary Figure S6: HER2 modulates CDK7 activity and CDK7-dependent gene transcription. a Top pathways of DE genes in HMEC-P13KCA^{H1047R} cells compared to the vector control HMEC-pBABE cells. **b** Top pathways of DE genes in pBABE-SHP2 WT and SHP2^{E76A} cells compared to the vector control HMEC-pBABE cells.

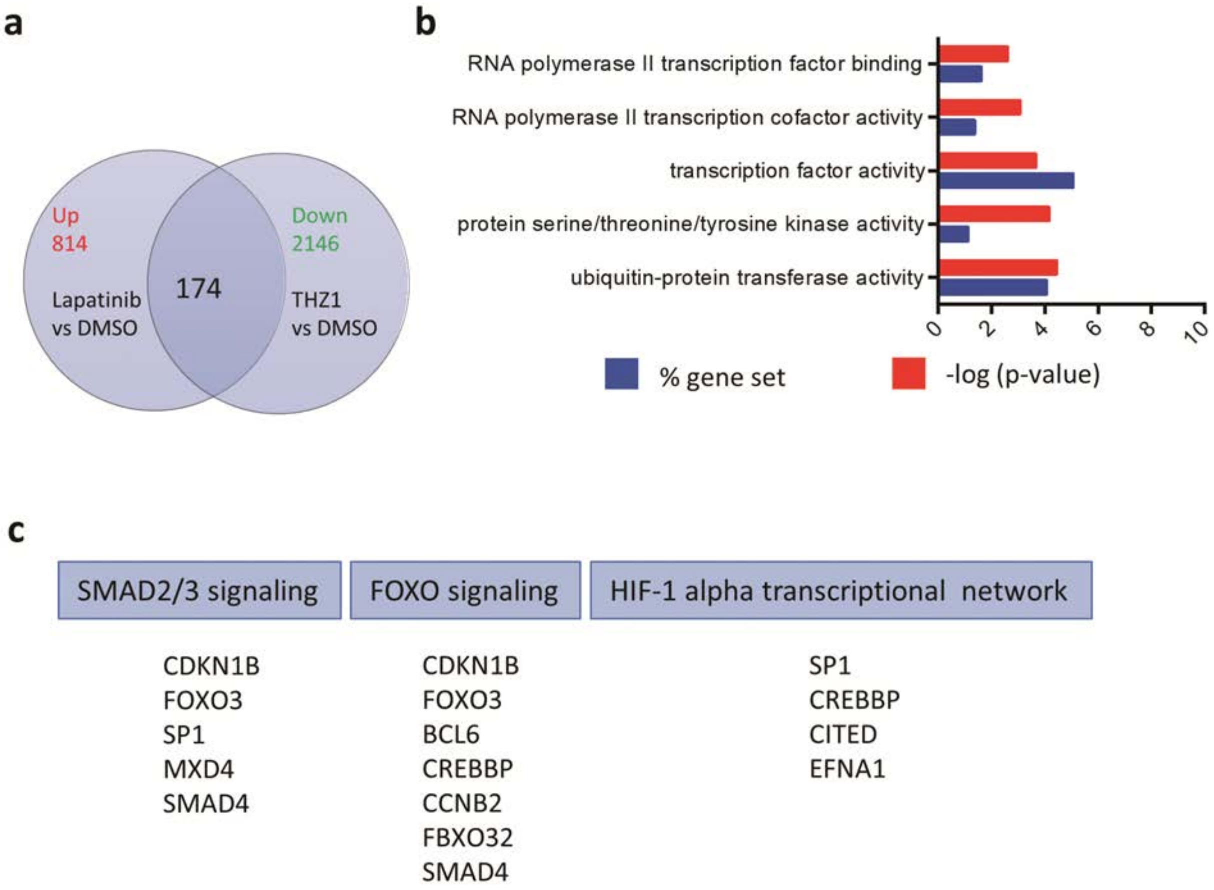

Supplementary Figure S7: CDK7 induces aberrant gene up-regulation that confers resistance to HER2 inhibitor. a Overlap between genes that were upregulated by lapatinib and those that were down-regulated by THZ1. **b** Enrichment for genes involved in regulation of transcription, kinase activity, and protein ubiquitination among genes upregulated by lapatinib. **c** Enrichment of genes involved in SMAD2/3 signaling, FOXO signaling, and HIF-1-alpha transcription factor network among genes upregulated by lapatinib.

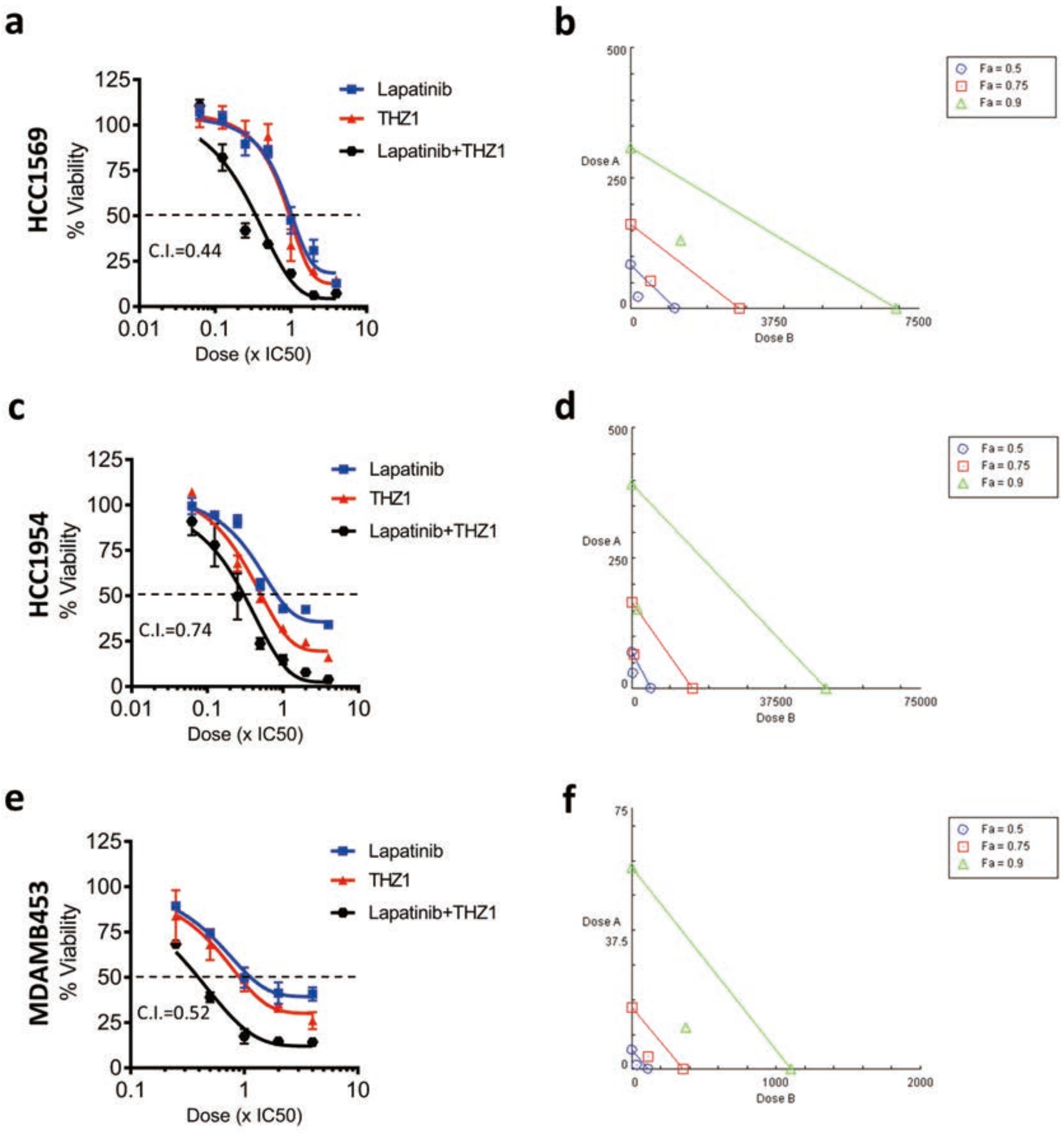

Supplementary Figure S8: THZ1 in combination with lapatinib enhances cell death in HER2iR BC cells. Cells were treated with the indicated combination of the drugs and analyzed as described in Methods and main Figure 5a. **a**, **c**, **e** Curve shift analysis. Degree of left shifted indicated the amount of synergism with the indicated drug-combination (blue, lapatinib; red, THZ1; black, both lapatinib and THZ1). **b**, **d**, **f** Isobolograms: The green, red

and blue lines indicate where the theoretical additive line is for a particular Fa value (here, blue, lapatinib; red, THZ1; green, both lapatinib and THZ1). The farther the calculated values far away from the origin, the greater the synergy between the drugs.

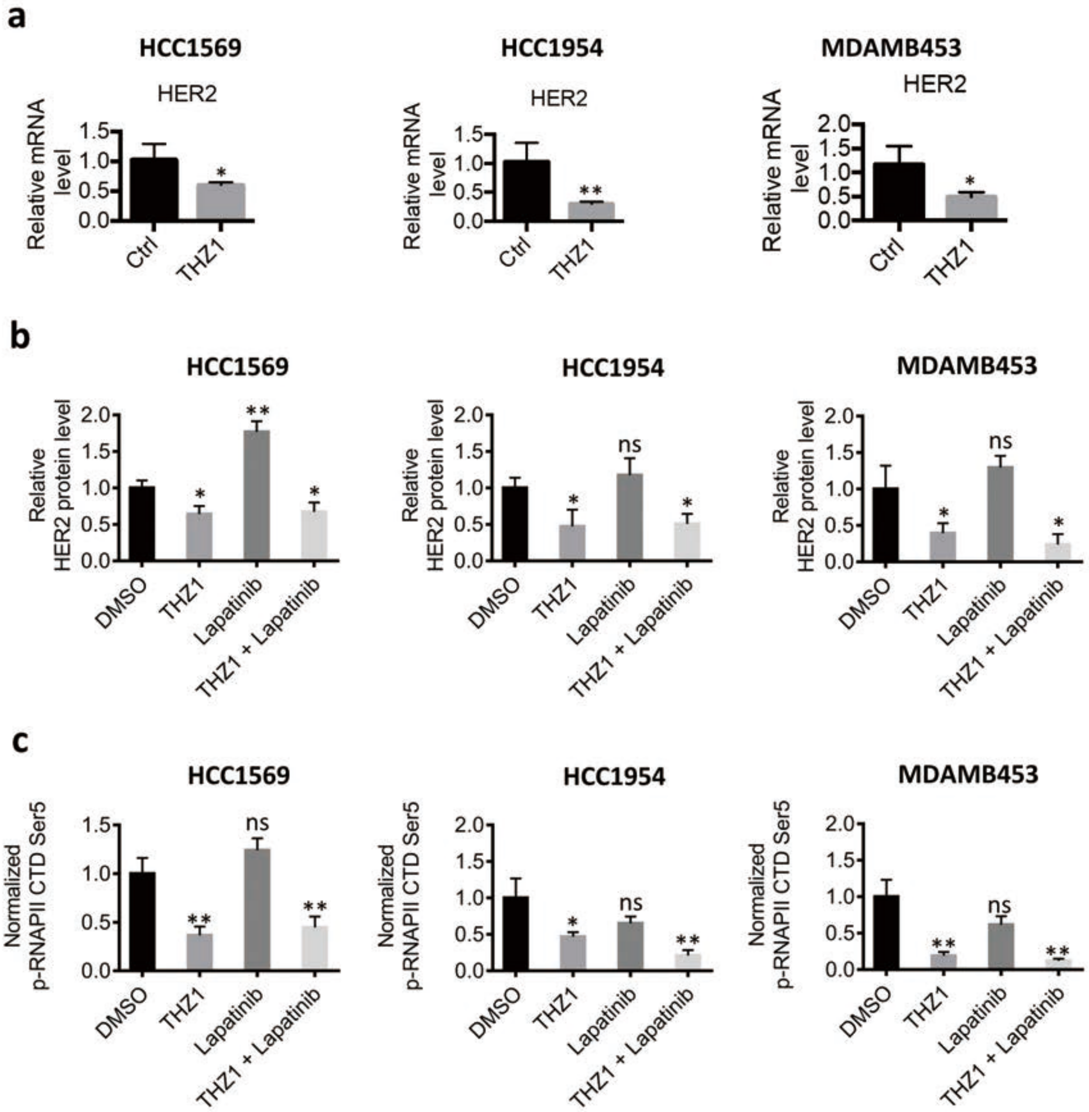

Supplementary Figure S9: Activation of CDK7/RNA Pol II cascade by pathways resistant to HER2 inhibition. a Cells were treated with THZ1 (250 nM) for 24 h. mRNA expression were determined using Q RT-PCR. Data represent mean ± SD (n= 3). *, $p < 0.05$; **, $p < 0.01$ (Student's *t* test). **b**, **c** Quantitation of HER2 and p-RNAPII CTD Ser5 expression in each cell line with or without inhibitor treatment, as described in Fig. 5d**.** Data represent

mean ± SD (n= 3). *, $p < 0.05$; **, $p < 0.01$; ***, $p < 0.005$; ns, not significant (Student's *t* test).

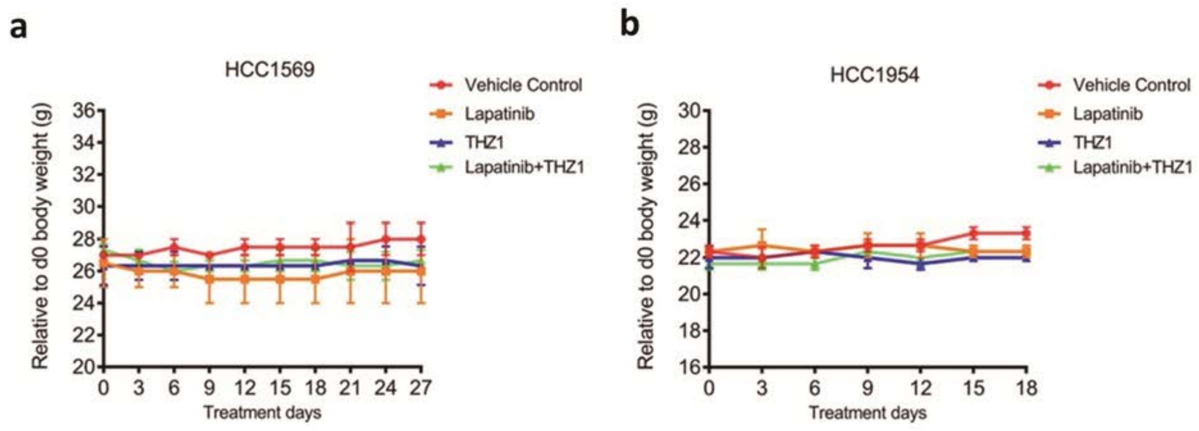

Supplementary Figure S10: Body weight of mice from each group in main Figure 6a, b. a, **b** Body weights of mice were measured as the indicated time of treatment. The mean weight for each group was shown as percentage change from day 0; error bars represent standard error.

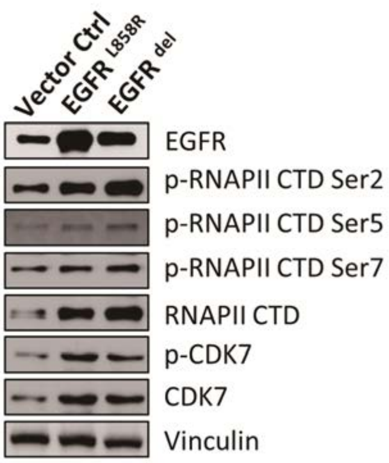

Supplementary Figure S11: Effect of ectopic expression of human EGFR1 mutants on activity of CDK7/RNA Pol II in HEMCs. Immunoblot analysis of activity and expression of CDK7/RNA Pol II complex in HMEC-EGFR mutant cell lines.

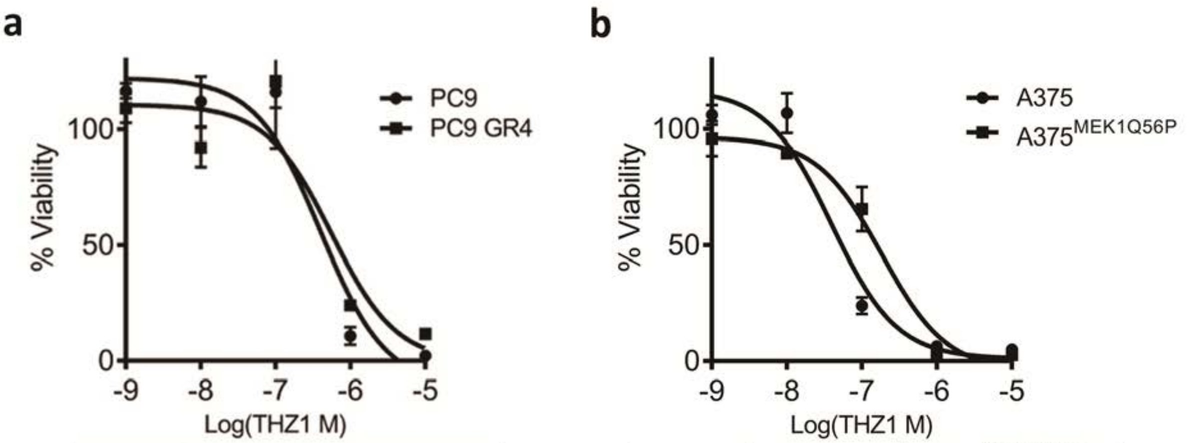

	PC9	PC9 GR4
IC50	4.042e-007	5.571e-007

	A375	A375^{MEK1Q56P}
IC50	3.946e-008	1.843e-007

Supplementary Figure S12: The effect of THZ1 in lung other cell model with additional kinase linage which confers resistance to the primary kinase inhibitor. a, **b** Dose-response curves of lung cancer and melanoma cell lines viability after treatment with increasing concentrations of THZ1 for 72 h. Percent viability relative to that of DMSO-treated cells were shown. Data represent mean ± SD of triplicates from three independent experiment.

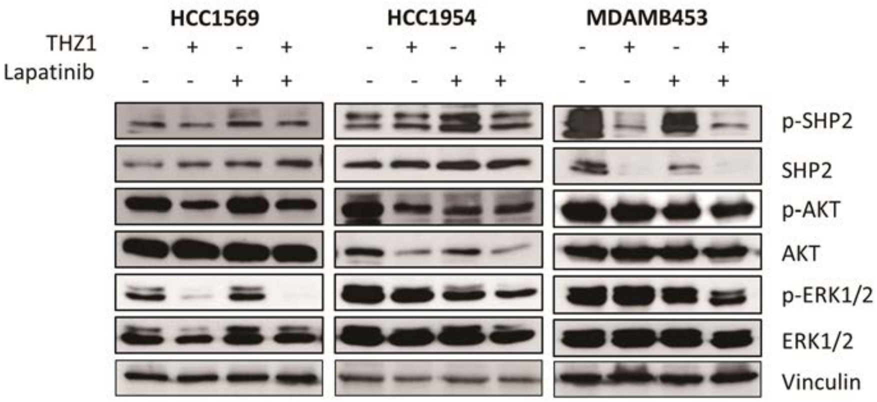

Supplementary Figure S13: HCC1569, HCC1954, and MDAMB453 cells were treated with vehicle control (DMSO), THZ1 (100 nM) and lapatinib (1 μM) alone or in combination for 24 h before immunoblotting using the indicated antibodies.

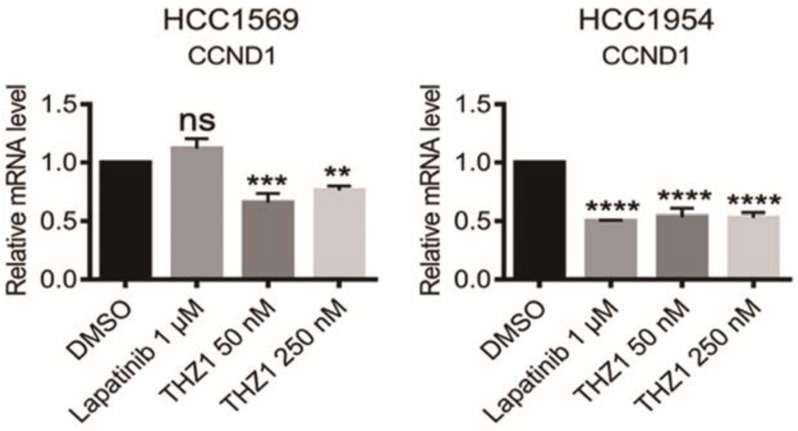

Supplementary Figure S14: Q-RT-PCR of CCND1 in HER2iR BC cells. Cells were treated with lapatinib (1 μM) or THZ1 (50 nM and 250 nM) for 24 h and the mRNA levels were determined using Q RT-PCR. Data represent mean ± SD (n= 3). **, *p* < 0.01; ****p*, < 0.001. *****p*, < 0.0001; ns, not significant (Student's *t* test).
